# Intercellular mitochondrial transfer and trans-mitophagy in response to protein import dysfunction

**DOI:** 10.1083/jcb.202511211

**Published:** 2026-07-07

**Authors:** Emily Glover, Beth Wiseman, Celyn Dugdale, Charlie Humphery, Lorena Sueiro Ballesteros, Lorna Hodgson, Kevin Wilkinson, Ian Collinson

**Affiliations:** 1 School of Biochemistry, University of Bristol, Bristol, UK; 2 School of Physiology, Pharmacology and Neuroscience, University of Bristol, Bristol, UK; 3 Flow Cytometry Facility, School of Cellular and Molecular Medicine, University of Bristol, Bristol, UK; 4 Wolfson Bioimaging Facility, University of Bristol, Bristol, UK

## Abstract

Mitochondrial protein import is critical for organelle biogenesis, maintenance, and regeneration—essential for cellular homeostasis. Import dysfunction compromises cellular energy supplies, which is damaging to cells, particularly those with high energetic demands like neurons. Previously, we have shown that import failure is rescued by intercellular mitochondrial transfer (IMT) via tunnelling nanotubes (TNTs) however, the fate of the transferred mitochondria and the mechanistic basis for rescue were unresolved. Here, we show that bidirectional mitochondrial trafficking between cells harboring import-defective and import-competent mitochondria is distinct in terms of their regulation and ensuing consequences. Transferred import-defective mitochondria are highly fragmented and destined for canonical lysosomal degradation. In contrast, reactive oxygen species (ROS)-producing mitochondria at the periphery of cells with import-competent mitochondria are transferred into neighboring cells undergoing import failure. These new arrivals then accumulate within previously uncharacterized “mitochondrial degradation bodies” (MDBs). We speculate that the cooperation of these distinct cases of TNT-mediated conventional and noncanonical “trans-mitophagy” instigates mitochondrial regeneration, and thereby rescues mitochondrial function.

## Teaser

Glover et al. show that cells subject to mitochondrial protein import failure induce IMT via tunnelling nanotubes. This rescue response involves the transfer of import-defective mitochondria into healthy cells for degradation; while functional, ROS-producing mitochondria are transferred into import-compromised cells and accumulate into novel organelles (MDB) for noncanonical trans-mitophagy.

## Introduction

Intercellular mitochondrial transfer (IMT) can have a significant and sustained beneficial impact on recipient cell metabolism (Baldwin et al., 2024; Islam et al., 2012; Lin et al., 2024; Hoover et al., 2025; Marlein et al., 2017; Marlein et al., 2019; Brestoff et al., 2021), even leading to rescue of cells containing defective mitochondria (Spees et al., 2006; Rostami et al., 2017; Needs et al., 2024). However, in most previous studies it is unclear how transferred mitochondria have been able to elicit these effects.

Exogenous mitochondria can have a diversity of fates, including incorporation into (Crewe et al., 2021) or the replacement of (Ikeda et al., 2025) the endogenous network, in addition to the segregation from the endogenous network as a functional mitochondrial subpopulation (Baldwin et al., 2024; Masuzawa et al., 2013). Surprisingly though, most studies show that exogenous mitochondria remain segregated from the endogenous network and display a small and spherical morphology (Lin et al., 2024; Islam et al., 2012; Kidwell et al., 2023; Vallabhaneni et al., 2012; Wang and Gerdes, 2015; Yao et al., 2018), sometimes clustered in the perinuclear region (Lin et al., 2024; Islam et al., 2012). Notably, these features are characteristic of lysosomal mitochondria (Frank et al., 2012; Pu et al., 2016), suggesting that they are destined for degradation.

Recent reports suggest that newly arrived mitochondria may function as instigators of signalling pathways in the receiving cell (Lin et al., 2024; Kidwell et al., 2023; Ikeda et al., 2025), explaining in part how small numbers of dysfunctional and short-lived exogenous mitochondria can induce profound cell-wide responses. Other reports describe a different narrative, whereby mitochondria are exported to neighboring cells in order to outsource their degradation—“trans-mitophagy” (Davis et al., 2014; Morales et al., 2020; Nicolás-Ávila et al., 2020)—for the purposes of quality control. In this scenario, the recipient cells reap no reward from this incursion, but merely act as mitochondrial trash cans (Nicolás-Ávila et al., 2020). It is unclear how trans-cellular mitochondrial degradation can result in two distinctive outcomes—signalling or quality control—and how these distinct functions might cooperate for the benefit of the recipient cell, such as the conferral of metabolic rescue.

Previously, we have shown that cells subject to chronic mitochondrial import failure upregulate TNT-mediated IMT and this rescues the import defect (Needs et al., 2024). However, the fate of exogenous mitochondria after transfer and the underlying mechanism of rescue was not determined. In the present study, we demonstrate bidirectional transfer between co-cultured cells containing import-defective and import-competent mitochondria. Following this two-way traffic, we deployed flow cytometry to isolate cells containing exogenous mitochondria. Fluorescence and electron microscopy shows that transferred import-defective mitochondria are exported for conventional mitophagy, while transferred import-competent mitochondria are sequestered into a membrane-bound “mitochondrial degradation body” (MDB) for noncanonical degradation. Our findings suggest that this trans-mitophagy has both mitochondrial quality control and signalling roles, which together rescue cells harboring import-defective mitochondria.

## Results and discussion

### TNT-mediated IMT in response to mitochondrial import failure is bidirectional

Previously, we have found that the number of TNTs increased in response to conditions of chronic mitochondrial import inhibition—induced for the mediation of IMT (Needs et al., 2023; Needs et al., 2024). However, the directionality of TNT-mediated mitochondrial transfer between cells containing import-defective mitochondria and cells containing import-competent mitochondria and their respective consequences have not been fully explored.

**Figure 1.**
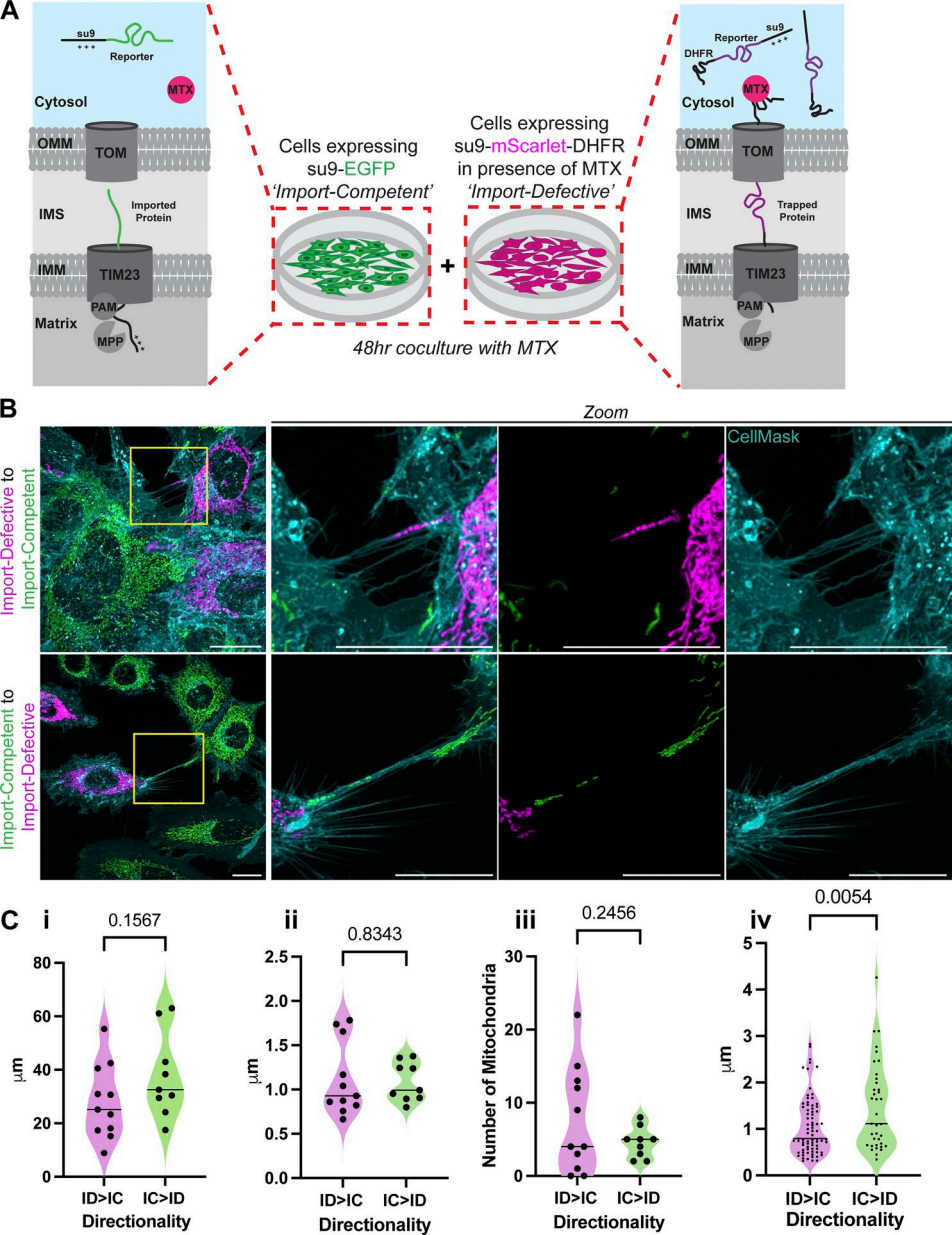
**TNT-mediated IMT in response to mitochondrial import failure is bidirectional. (A)** Schematic of the co-culture system. **(B)** Top panel: Representative image of su9-mScarlet-DHFR (+MTX)-expressing mitochondria (import-defective, magenta) within a TNT. Bottom panel: Representative image of su9-EGFP-expressing mitochondria (import-competent, green) within a TNT. Scale bars 20 μm. **(C)** Quantification of 20 TNTs over *N* = 3 technical repeats of (i) TNT length, (ii) average TNT width, (iii) number of mitochondria in TNT, and (iv) length of each individual mitochondrion within TNTs. P values from unpaired two-tailed *t* tests. Mean lines shown on the graph. ID, import-defective; IC, import-competent.

To address these questions, HeLa cells were conditioned in galactose (HeLaGAL), rendering cells OXPHOS-dependent and thus sensitive to mitochondrial perturbation (Aguer et al., 2011; Marroquin et al., 2007). HeLaGAL cell lines were then created with stable expression of a precursor construct comprising the powerful mitochondrial targeting sequence of subunit nine of the ATP synthase from the fungus *Neurospora crassa* (su9) (Westermann and Neupert, 2000; Hartl et al., 1989), fused to the fluorescent proteins EGFP, mScarlet, or mTagBFP2 for visualization. Dihydrofolate reductase (DHFR) was fused to the C terminus of this precursor to create lines susceptible to mitochondrial import inhibition by virtue of DHFR resisting unfolding when bound to the small molecule methotrexate (MTX). Given that import requires precursor unfolding, this blocks the import machinery (Rassow et al., 1989; Eilers and Schatz, 1986; Needs et al., 2024) (Fig. 1 A).

To investigate the TNT-mediated transfer of import-defective versus import-competent mitochondria, su9-mScarlet-DHFR and su9-EGFP lines were co-cultured with MTX for 48 h (Fig. 1 A). Live fluorescence imaging of mitochondria-containing TNTs (mitoTNTs), identified as a membranous tube containing mitochondria connecting a su9-mScarlet-DHFR–expressing cell and a su9-EGFP–expressing cell, revealed similar numbers of mitoTNTs containing import-defective mitochondria (55%) and mitoTNTs containing import-competent mitochondria (45%) (Fig. 1 B). This indicates that both import-competent and import-defective mitochondria are mobilized. Notably, import-defective and import-competent mitochondria were never observed in the same TNT at the same time, suggesting that mitochondrial transfer within an individual TNT is unidirectional. Irrespective of the state of the transiting mitochondria, mitoTNT length and width were similar (Fig. 1, C i and ii).

With respect to the frequency of the mitochondrial traffic within each TNT, there tended to be more import-defective mitochondria within each individual (mean of 7.55) in comparison with import-competent mitochondria (mean of 4.56) (Fig. 1 C iii). Transiting import-defective mitochondria were significantly shorter than those that were import-competent (1.05 and 1.47 μm, respectively) (Fig. 1 C iv), consistent with the former being more prone to fission (Needs et al., 2024). Taken together, these data suggest that intercellular transfer of import-competent and import-defective mitochondria both make significant contributions to the rescue of chronic import inhibition previously observed (Needs et al., 2024).

### Cells subject to IMT can be isolated by fluorescence-activated cell sorting

Flow cytometry approaches can be used to quantify and isolate cells that have received intercellularly transferred cargo (Kidwell et al., 2023; Ikeda et al., 2025; Lin et al., 2024; Kolba et al., 2019; Sharma and Subramaniam, 2019; Kumar et al., 2017). Therefore, we developed a flow cytometry assay in which import-defective and import-competent mitochondria can be distinguished by different fluorescently labeled mitochondria. Cells subject to IMT could thus be identified and isolated by fluorescence-activated cell sorting (FACS) for analysis.

**Figure S1.**
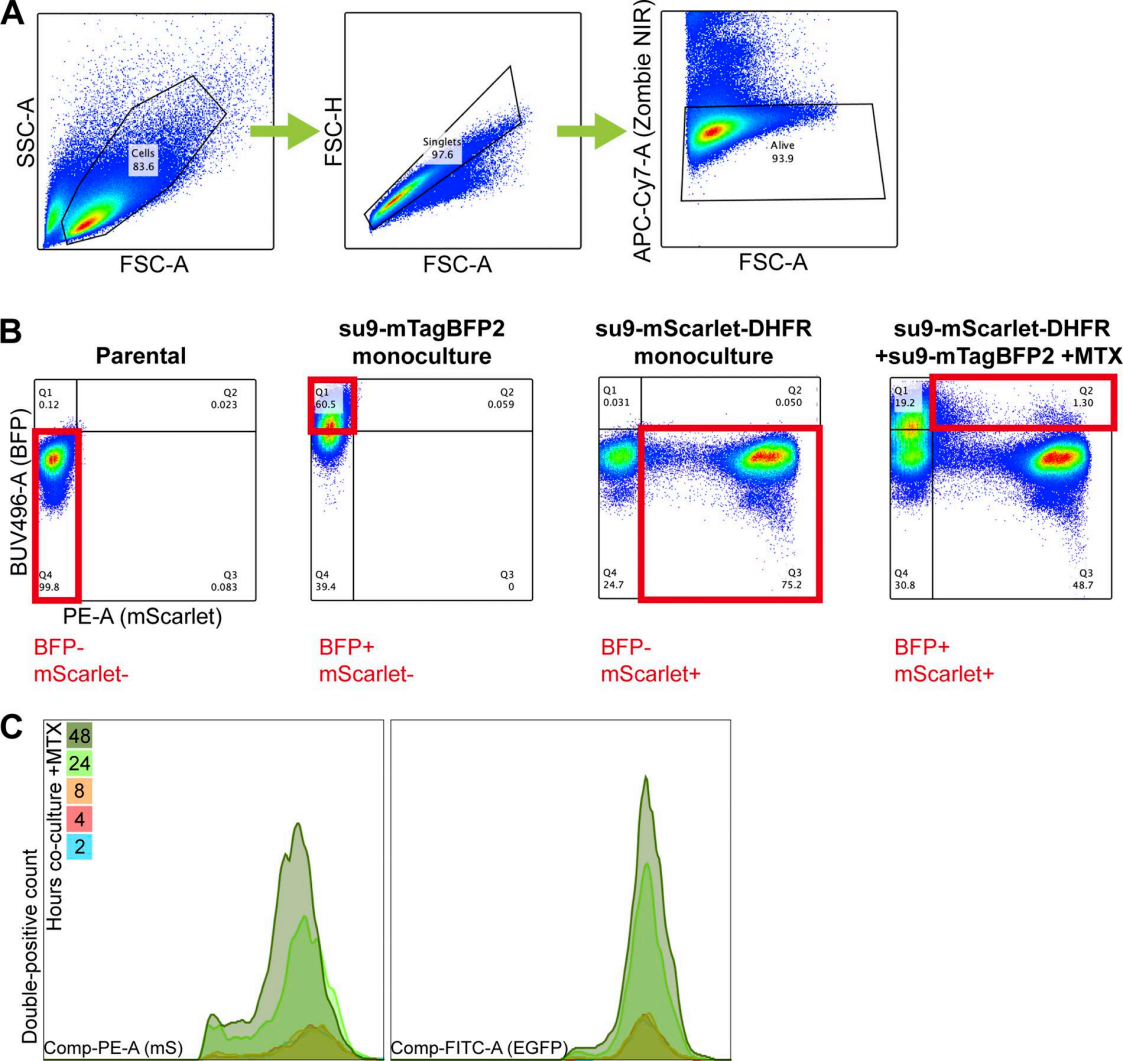
**Cells subject to IMT can be isolated by FACS. (A)** Gating strategy for isolation of cells that are singlets and alive. **(B)** Four-way gating system for identification of BFP+/mScarlet+ cells. **(C)** Representative histograms of double-positive cell count vs. mScarlet or EGFP intensity for each time point of co-cultivation time course. Q; quartile. SSC-A; side scatter area. FSC-A; forward scatter area. Comp; compensation.

**Figure 2.**
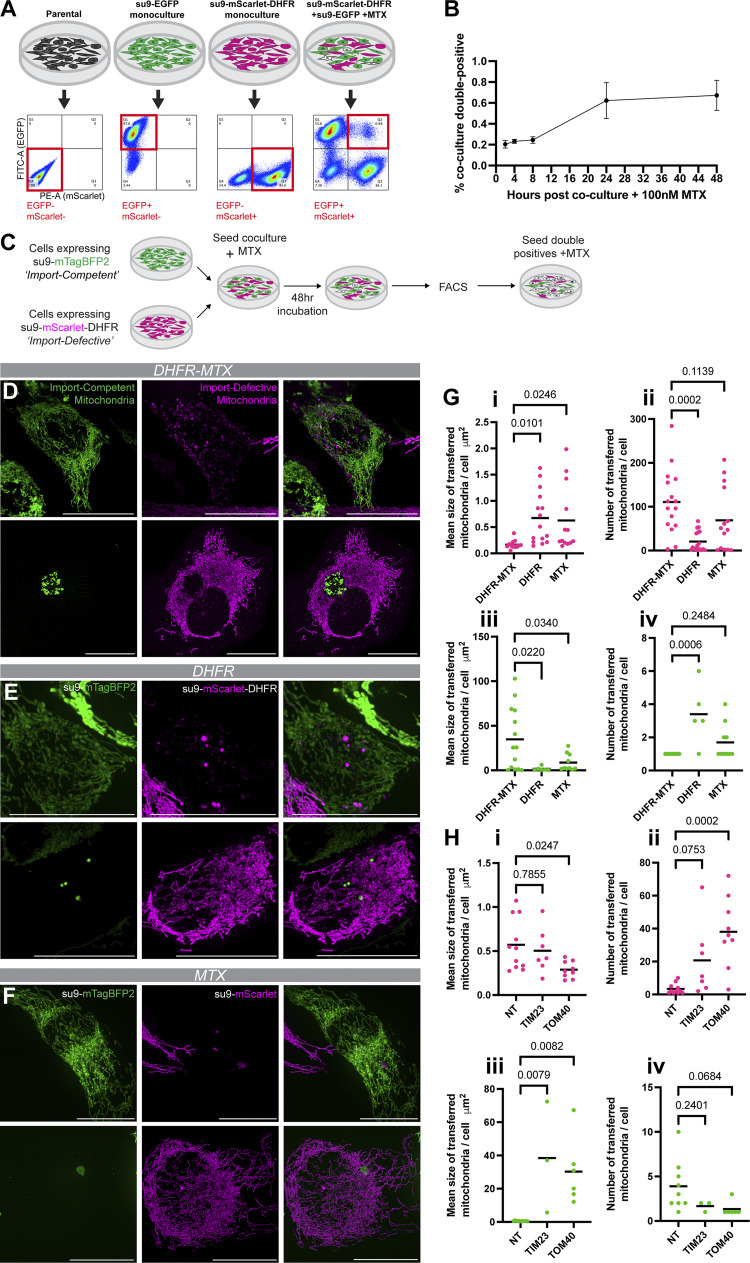
**Transferred import-competent and import-defective mitochondria have distinct fates. (A)** Four-way gating system for identification of EGFP+/mScarlet+ cells. **(B)** Time course of the percentage population of co-culture that is double-positive for EGFP and mScarlet in a co-cultivation of su9-EGFP line (import-competent) with su9-mScarlet-DHFR line in the presence of MTX (import-defective). *N* = 4 technical replicates. Error bars represent standard deviation. **(C)** Schematic of workflow. **(D)** Representative images of double-positive cells from a 48 h co-culture of su9-mTagBFP2 and su9-mScarlet-DHFR cell lines in the presence of MTX. **(E)** Representative images of double-positive cells from a 48 h co-culture of su9-mTagBFP2 and su9-mScarlet-DHFR cell lines. **(F)** Representative images of double-positive cells from a 48 h co-culture of su9-mTagBFP2 and su9-mScarlet cell lines in the presence of MTX. Scale bars 20 μm. **(G)** Quantification of (i) mean size of transferred import-defective mitochondria per cell; (ii) number of transferred import-defective mitochondria per cell; (iii) mean size of transferred import-competent mitochondria per cell. For transferred import-competent mitochondria localized in MDBs, individual mitochondria could not be distinguished, so the total MDB area was quantified; (iv) number of transferred import-competent mitochondria per cell. All quantification was done on max. projected images. *n* = 62 cells from *N* = 3 technical replicates. P values from ordinary one-way ANOVA, multiple comparisons corrected for by the Dunnett test. Data distribution was assumed to be normal, but this was not formally tested. Mean lines shown on the graph. **(H)** Quantification of double-positive cells from a 48 h co-culture of su9-mTagBFP2 line and a su9-mScarlet line, in which the su9-mScarlet line has been pre-treated with siRNA against TIM23, TOM40, or a non-targeting control. (i) mean size of transferred import-defective mitochondria per cell; (ii) number of transferred import-defective mitochondria per cell; (iii) mean size of transferred import-competent mitochondria per cell. For transferred import-competent mitochondria localized in MDBs, individual mitochondria could not be distinguished, so the total MDB area was quantified; (iv) number of transferred import-competent mitochondria per cell. All quantification was done on max. projected images. *n* = 45 cells from *N* = 3 technical replicates. P values from ordinary one-way ANOVA, multiple comparisons corrected for by the Dunnett test. Data distribution was assumed to be normal, but this was not formally tested. Mean lines shown on the graph.

Following gating of alive singlet cells using parental HeLaGALs (Fig. S1 A), a four-way gating system was set up using su9-mScarlet-DHFR and su9-EGFP mono-cultures (Fig. 2 A). A co-culture of su9-mScarlet-DHFR and su9-EGFP stable cell lines with MTX was then subject to this gating strategy.

The majority of live singlet cells in this co-culture were separated into either the EGFP only or mScarlet only gates, representing cells that have not received exogenous mitochondria. However, a population of cells was double-positive, containing both EGFP and mScarlet, indicative of their containment of both import competent and compromised mitochondria—the result of mitochondrial transfer (Fig. 2 A). Analysis after different times of co-cultivation showed that this double-positive population increases over time (Fig. 2 B).

Given the possibility of lysosomal encapsulation for degradation (mitophagy) of migrating mitochondria and the resulting low pH, it became necessary to switch to a pH-insensitive fluorophore to monitor their fate. Therefore, EGFP, which is quenched in the acidic environment of the lysosome, was exchanged for mTagBFP2, which is not (Kim and Seong, 2021; Shinoda et al., 2018). Importantly, for the purposes of our analysis, mTagBFP2 is subject to lysosomal proteolysis (Shinoda et al., 2018), while mScarlet is neither quenched nor degraded (Clancy et al., 2023). Therefore, lysosomal degradation of mitochondria could be characterized by the gradual loss of mTagBFP2 fluorescence.

A four-way gating system analogous to that used above was set up using su9-mScarlet-DHFR and su9-mTagBFP2 mono-cultures (Fig. S1 B). Our analysis showed this was capable, albeit at a slight loss in resolution, of distinguishing cells containing both mTagBFP2 and mScarlet for sorting.

### Transferred import-competent and import-defective mitochondria have distinct fates

To investigate the consequences of mitochondrial transfer, a co-culture of su9-mScarlet-DHFR– and su9-mTagBFP2–expressing cell lines were grown in the presence of MTX. Cells that were double-positive for mScarlet and mTagBFP2 were then isolated by FACS for live fluorescence imaging (Fig. 2 C).

Interestingly, two distinctive types of cells were observed (Fig. 2 D). In the first of these, cells containing import-competent mitochondria (su9-mTagBFP2) were the recipients of numerous (mean of 111) import-defective mitochondria (su9-mScarlet-DHFR), which were small (mean projected area of 0.163 µm^2^), spherical, and dispersed throughout the cell (Fig. 2 D, top panel). The second type was cells containing a network of import-defective mitochondria (su9-mScarlet-DHFR) with the addition of a defined cluster of import-competent mitochondria (su9-mTagBFP2)—referred to hereafter as a mitochondrial degradation body (MDB) (Fig. 2 D, bottom panel).

**Figure S2.**
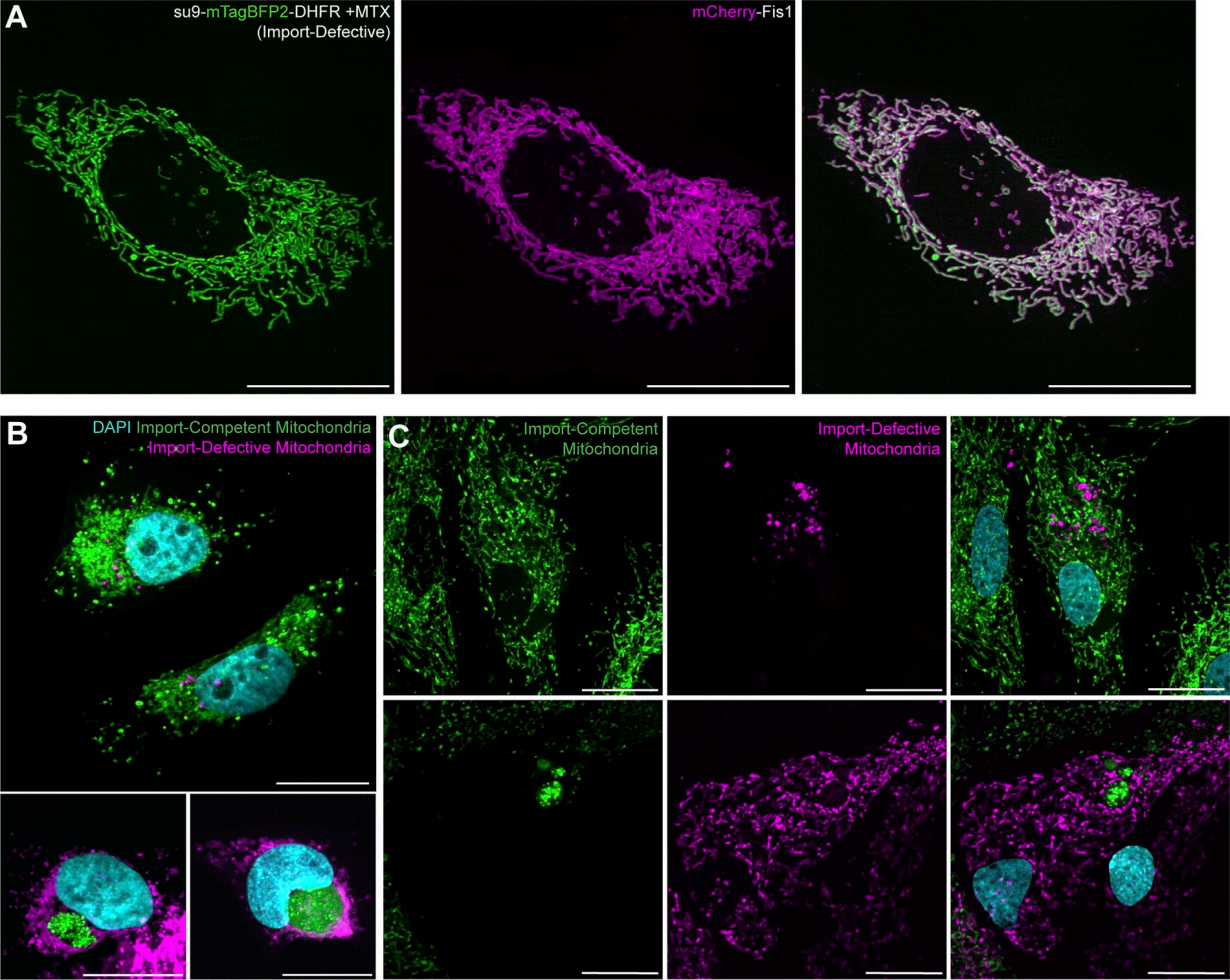
**Fis1, DAPI, and astrocytes. (A)** Representative image of su9-mTagBFP2-DHFR cell line following 48 h incubation with MTX (import-defective) and transfection with mCherry-Fis1, showing co-localization. **(B)** Representative images of double-positive cells from a 48 h co-culture of su9-EGFP line (import-competent) and su9-mScarlet-DHFR line in the presence of MTX (import-defective) following fixation and DAPI staining. **(C)** Examples of double-positive cells from a co-culture of primary astrocytes expressing either cox8a-DsRed (import-competent) or su9-EGFP-DHFR in the presence of MTX (import-defective). Scale bars 20 μm.

On average, a singular extraordinarily large MDB (mean projected area of 34.8 µm^2^) could be seen in each import-defective cell. We confirmed that su9-mTagBFP2-DHFR is indeed specifically localized to mitochondria following 48 h incubation with MTX (Fig. S2 A), validating that the transferred fluorescent protein corresponds to imported mitochondria, and thus is a *bona fide* measure of IMT (rather than of non-mitochondrial material). In addition, DAPI staining confirmed that these MDBs were devoid of chromatin and, therefore, unlikely to have arisen by cell–cell fusion and consistent with them being the product of IMT (Fig. S2 B). Furthermore, we observed analogous structures in a co-culture of import-competent rat primary astrocytes (cox8a-DsRed) alongside those with import-defective mitochondria (su9-EGFP-DHFR) (Fig. 2 C). Therefore, these distinctive MDBs are unlikely to be an artefact of the clonal cell lines examined thus far.

Taken together, the results show that IMT occurs bidirectionally between cells populated by import-competent and import-defective mitochondria. Additionally, the transferred mitochondria do not fuse and become incorporated into the recipient mitochondrial network, consistent with what has been observed elsewhere (Lin et al., 2024; Ikeda et al., 2025; Islam et al., 2012; Kidwell et al., 2023; Vallabhaneni et al., 2012; Wang and Gerdes, 2015; Yao et al., 2018; Baldwin et al., 2024). While the appearance of transferred import-defective mitochondria within the recipient is typical of the small and spherical morphology of transferred mitochondria seen previously (Kidwell et al., 2023; Vallabhaneni et al., 2012; Wang and Gerdes, 2015; Yao et al., 2018; Lin et al., 2024; Islam et al., 2012), the appearance of MDBs upon the counterflow of import-competent mitochondria was entirely unexpected.

### IMT and the fate of transferred mitochondria are dependent on the loss of protein import activity

To verify that these effects were the consequences of mitochondrial import inhibition, the impacts of DHFR or MTX removal from the co-culture were assessed. Cells expressing su9-mTagBFP2 were co-cultured together with either (1) su9-mScarlet-DHFR in the absence of MTX (Fig. 2 E) or (2) su9-mScarlet with MTX (Fig. 2 F). Double-positive cells were then isolated and compared with those from su9-mTagBFP2 and su9-mScarlet-DHFR co-cultures in the presence of MTX.

With regards to transferred mScarlet-labelled mitochondria, analysis showed that their small size (mean projected volume of 0.163 µm^2^) was dependent on blocked import sites (presence of DHFR and MTX), since DHFR or MTX alone resulted in a larger size (mean projected volumes of 0.671 and 0.626 µm^2^, respectively) (Fig. 2 G i). Moreover, import-defective mitochondria have a higher frequency of mitochondrial transfer: on average 111/cell compared with DHFR (20/cell) or MTX (69/cell) utilised alone (Fig. 2 G ii).

With respect to transferred import-competent mitochondria (visualized by mTagBFP2), their accumulation into large MDBs, characterized by low numbers and very large size, was dependent on their entry into cells subject to mitochondrial import site blocking (presence of DHFR and MTX) (Fig. 2, G iii–iv). When the recipients were exposed to a DHFR-containing precursor or MTX separately, the transferred import-competent mitochondria were less inclined to be segregated into MDBs, as demonstrated by their larger number and smaller size (Fig. 2, G iii–iv).

Therefore, IMT still occurs in co-cultures wherein both cell lines contain import-competent mitochondria (DHFR or MTX omitted). However, the transfer of a large number of highly fragmented mitochondria as well as the trafficking of mitochondria into MDBs are specific features of bidirectional transfer between cells with import-competent mitochondria and cells undergoing mitochondrial import failure.

**Figure S3.**
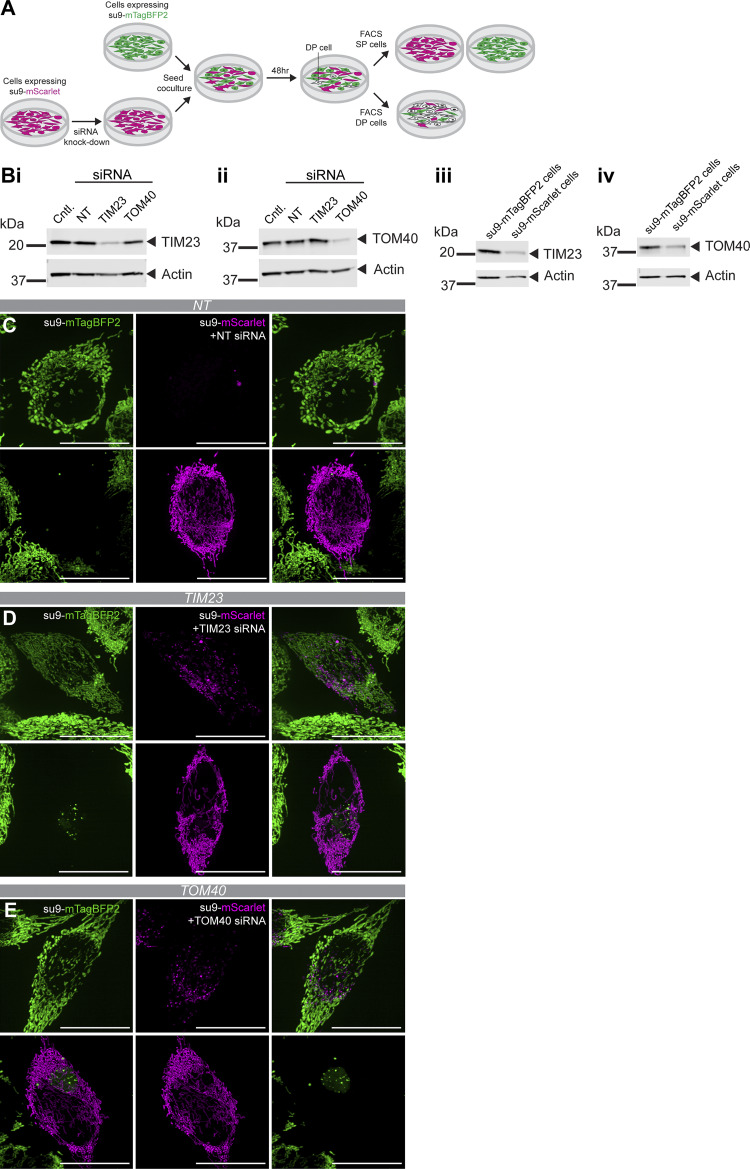
**TIM23 and TOM40 siRNA knockdowns recapitulate phenotypes seen with DHFR-MTX. (A)** Schematic of workflow. **(B)** Western blots of (i) TIM23 siRNA knockdown; (ii) TOM40 siRNA knockdown; (iii) FACS-isolated single-positive cells following TIM23 siRNA knockdown in su9-mScarlet cell line and subsequent 48 h co-culture with su9-mTagBFP2 cell line; (iv) FACS-isolated single-positive cells following TOM40 siRNA knockdown in su9-mScarlet cell line and subsequent 48 h co-culture with su9-mTagBFP2 cell line. **(C)** Representative images of double-positive cells from a 48 h co-culture of a su9-mTagBFP2 line with a non-targeting siRNA-treated su9-mScarlet line. **(D)** Representative images of double-positive cells from a 48 h co-culture of a su9-mTagBFP2 line and a TIM23 siRNA-treated su9-mScarlet cell line. **(E)** Representative images of double-positive cells from a 48 h co-culture of a su9-mTagBFP2 line and a TOM40 siRNA-treated su9-mScarlet cell line. Scale bars 20 μm. DP, double-positive cell; SP, single-positive cell; Cntl, control; NT, non-targeting. Source data are available for this figure: SourceData FS3.

Next experiments were established to assess whether these phenotypes were conserved consequences of impaired mitochondrial protein import inhibition, as opposed to a specific feature of the MTX–DHFR system. To do so, a su9-mScarlet line was treated with siRNA to deplete the mitochondrial import machinery: against TOM40 or TIM23 (or a control non-targeting siRNA). The resultant cells were then co-cultured with a su9-mTagBFP2 line (Fig. S3 A) for 48 h prior to FACS of cells double-positive for mScarlet and mTagBFP2. Western blotting confirmed the proteins were knocked down for the duration of the experiment (Fig. S3, B i–iv). Double-positive cells subject to live fluorescence imaging recapitulated the phenotypes observed with the MTX–DHFR system (Fig. S3, C–E).

In regard to transferred mScarlet-labelled mitochondria, analysis showed that their small size was also brought about when essential components of import machinery of the outer and inner membranes were reduced. In the former, TOM40 knockdowns resulted in a mean projected area of 0.289 µm^2^, compared with non-targeting siRNA, which resulted in a larger size (mean projected area of 0.571 µm^2^) (Fig. 2 H i). In the latter case, the reduced size was marginal when TIM23 was knocked down (0.503 µm^2^; Fig. 2 H i).

In addition to the effects on mitochondrial size, import-defective mitochondria also possess a higher frequency of IMT: on average 21/cell and 38/cell for TIM23 and TOM40 knockdowns respectively, compared with non-targeting siRNA (3/cell) (Fig. 2 H ii).

With respect to transferred import-competent mitochondria (visualized by mTagBFP2), their accumulation into large MDBs was also dependent on their entry into cells subject to mitochondrial import inhibition (in this case, TIM23 or TOM40 knockdown) (Fig. 2, H iii–iv). When the recipients were exposed to the non-targeting siRNA control, the transferred import-competent mitochondria did not segregate into MDBs, as demonstrated by their smaller size.

Interestingly, both double-positive phenotypes were generally more pronounced in response to TOM40 knockdown in comparison with TIM23 knockdown, perhaps reflecting the greater impact of a decrease in the operation of the TOM complex over the TIM23 complex. This is likely because the TOM complex is critical for all aspects of mitochondrial protein import- to the outer membrane, inter membrane space, inner-membrane, and the matrix - whereas the TIM23 complex is concerned only with matrix import (Neupert and Herrmann, 2007). Furthermore, protein translocation during import is rate-limiting through the outer membrane but unrestricted through the inner membrane (Ford et al., 2022), consistent with the bigger effect of TOM40 depletion.

### Transferred import-defective and import-competent mitochondria undergo distinct forms of trans-mitophagy

**Figure 3.**
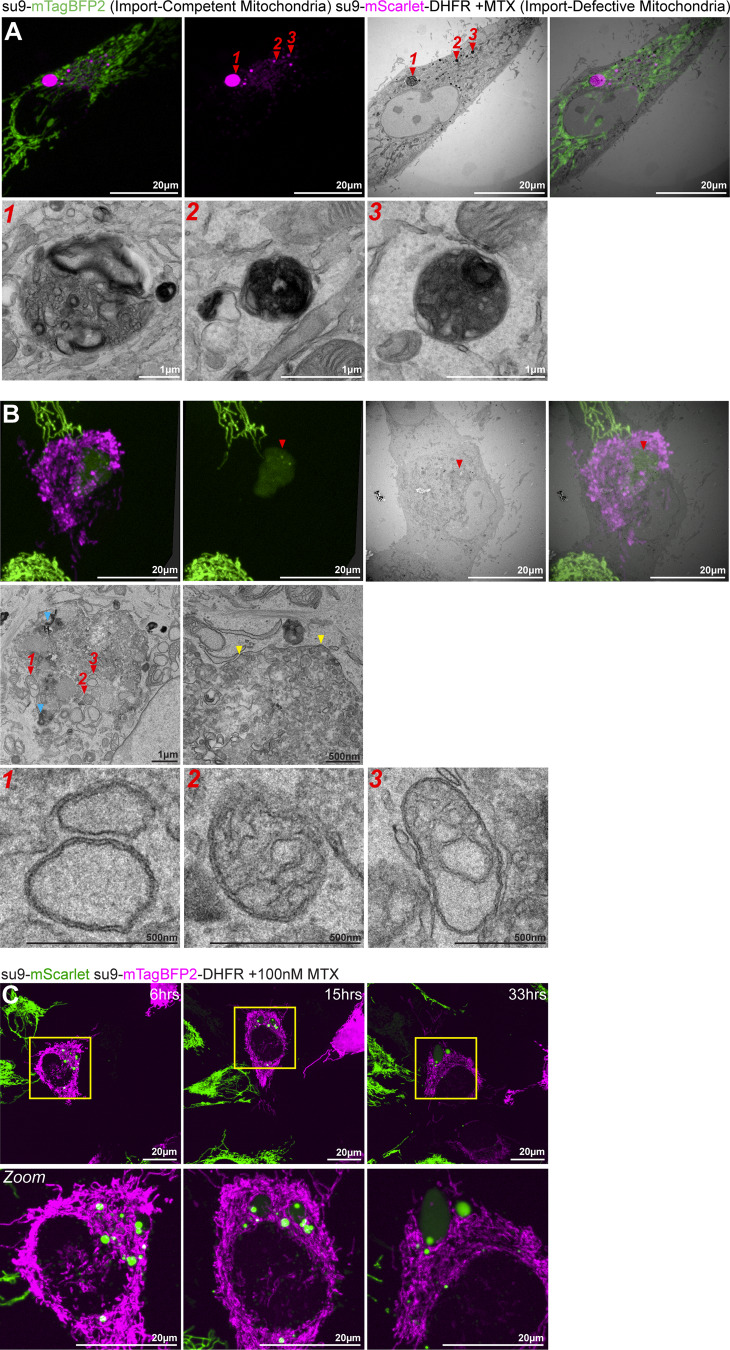
**Transferred import-defective and import-competent mitochondria undergo distinct forms of trans-mitophagy. (A)** Top panel: Fluorescence microscopy image of transferred import-defective mitochondria (red arrows) correlated with the electron microscopy image. Bottom panel: Electron microscopy images of the indicated transferred lysosomal import-defective mitochondria. **(B)** Top panel: Fluorescence microscopy image of MDB (red arrow) correlated with the electron microscopy image. Middle panel: Electron microscopy of MDB containing potential ex-mitochondria (red arrows), lysosomes (blue arrows), and surrounded by a partial membrane (yellow arrows). Bottom panel: Electron microscopy images of indicated ex—import-competent mitochondria in MDB. **(C)** Stills from a movie of transferred import-competent mitochondria (su9-mScarlet) in a cell containing import-defective mitochondria (su9-mTagBFP2-DHFR +100 nM MTX) at the indicated time points after sorting, demonstrating MDB formation.

To further interrogate the fate of transferred mitochondria, we used correlative light and electron microscopy (CLEM). This showed that transferred import-defective mitochondria have a classical lysosomal morphology, indicative of conventional trans-mitophagy (Fig. 3 A, red arrows). In contrast, MDBs resulting from the transfer of import-competent mitochondria into cells undergoing mitochondrial import failure are packed with membranous material (Fig. 3 B). Double membrane-bound structures can be seen within (Fig. 3 B, red arrows), which are presumably mitochondria undergoing lysosomal degradation (Fig. 3 B, blue arrows). Furthermore, an encapsulating membrane is evident at the edge of the MDBs (Fig. 3 B, yellow arrows).

**Figure 4.**
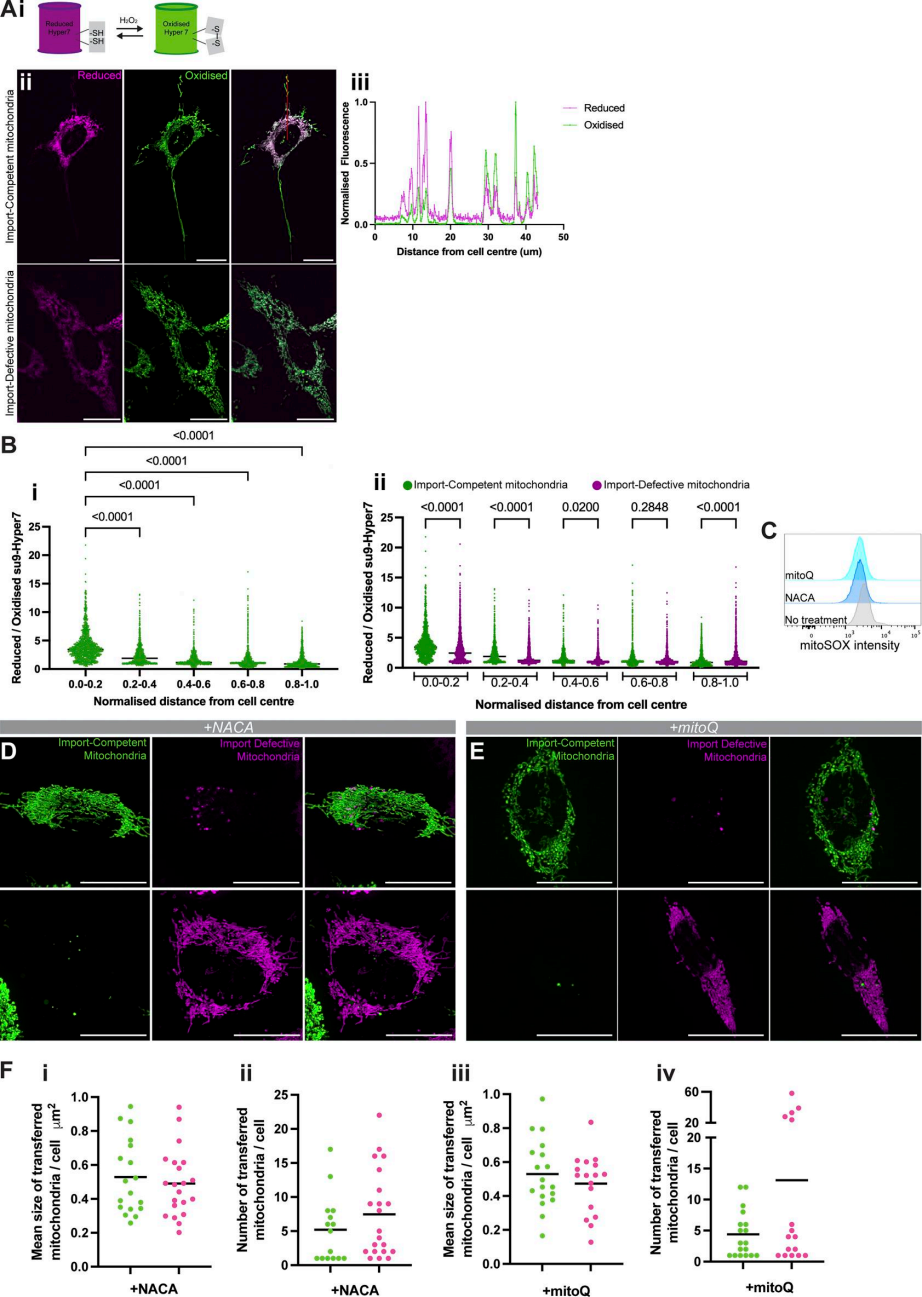
**ROS production** **dictates the fate of incoming mitochondria** **.** **(Ai)** Schematic of Hyper7 oxidation by hydrogen peroxide. **(Aii)** Representative image of su9-Hyper7-expressing cell line (top panel, import-competent mitochondria) and su9-Hyper7;su9-mScarlet-DHFR-expressing cell line in the presence of 100 nM MTX (bottom panel, import-defective mitochondria). **(Aiii)** normalized fluorescence intensities taken along the red line marked on import-competent cell in Aii of emission band pass (BP) 525/50 in response to excitation at 405 nm (reduced Hyper7) or excitation at 488 nm (oxidized Hyper7) vs. distance from cell center. **(Bi)** reduced/oxidized su9-Hyper7 ratio in HeLaGAL cell line vs. normalized distance from cell center. *n* = 17 cells over *N* = 3 technical repeats. Mean lines shown on the graph. **(Bii)** reduced/oxidized su9-Hyper7 ratio in HeLaGAL cell line (import-competent mitochondria) in comparison with HeLaGAL cell line transfected with su9-mScarlet-DHFR and treated with 100 nM MTX for 48 h (import-defective mitochondria) vs. normalized distance from cell center. Error bars represent standard deviation. *n* = 17–19 cells over *N* = 3 technical repeats. P values from one-way ANOVA, multiple comparisons corrected for by the Dunnett test. Data distribution was assumed to be normal, but this was not formally tested. **(C)** mitoSOX intensity of HeLaGAL cells in response to 48 h treatment with 750 μM NACA or 100 nM mitoQ in comparison with no treatment. **(D)** Representative images of double-positive cells from a 48 h co-culture of su9-mTagBFP2 and su9-mScarlet-DHFR cell lines in the presence of MTX and NACA. **(E)** Representative images of double-positive cells from a 48 h co-culture of su9-mTagBFP2 and su9-mScarlet-DHFR cell lines in the presence of MTX and mitoQ. **(F)** Quantification of (i) mean size of transferred mitochondria per cell with NACA addition; (ii) number of transferred mitochondria per cell with NACA addition; (iii) mean size of transferred mitochondria per cell with mitoQ addition; (iv) number of transferred mitochondria per cell with mitoQ addition. *n* = 40 cells from *N* = 3 technical replicates. Mean lines shown on the graph. Scale bars 20 μm. All quantification was done on max. projected images.

To gain time-resolved information regarding mitochondrial incorporation into MDBs, we performed live imaging on double-positive cells isolated from a su9-mScarlet and su9-mTagBFP2-DHFR co-culture in the presence of MTX. This showed that transferred import-competent mitochondria were initially present with a fragmented appearance analogous to those of import-defective mitochondria that have moved in the other direction (Fig. 4 C, 6 h time point). Then, over time, they cluster into increasingly large MDBs (Fig. 4 C, 33 h time point) in a process presumably instigated by the receiving cell undergoing mitochondrial import failure.

### ROS production dictates the fate of incoming mitochondria

Our observations so far pose the question of why transferred import-competent mitochondria are trafficked into MDBs, while transferred import-defective mitochondria remain dispersed. To assess the potential role of ROS in this distinction, we analyzed HeLaGAL containing the mitochondrial-targeted ROS-sensor HyPer7 (Pak et al., 2020) (Fig. 4 A i). Interestingly, we observed that peripheral mitochondria in import-competent HeLaGAL cells have a significantly more oxidized mitochondrial matrix relative to those in the perinuclear region (representative image in Fig. 4 A ii, top panel and quantification in iii and B). This distribution places oxidized mitochondria in a prime position for IMT. Notably, Kidwell et al. show that ROS production by transferred mitochondria can activate ERK signalling in the receiving cell (Kidwell et al., 2023). Therefore, we propose that ROS production by mitochondria transferred from the periphery of import-competent cells into those undergoing mitochondrial import failure promotes their trafficking into MDBs for noncanonical trans-mitophagy in the receiving cell.

In contrast, while perinuclear mitochondria in HeLaGAL cells harboring import-defective mitochondria present with a more oxidized matrix, the peripheral mitochondria in these cells are more reduced compared with HeLaGAL cells containing non-compromised organelles (representative image in Fig. 4 A ii, bottom panel and quantification in B). We suggest that the export of these mitochondria with relatively lower ROS production leads to more conventional lysosomal mediated mitophagy, rather than by the formation of MDBs.

To support this idea, we treated co-cultures of su9-mTagBFP2 cells (import-competent mitochondria) and su9-mScarlet-DHFR cells in the presence of MTX (import-defective mitochondria) with the antioxidants N-acetylcysteine amide (NACA) or mitoquinone (mitoQ). NACA is a general antioxidant (Sunitha et al., 2013) and mitoQ is a mitochondria-targeted antioxidant (Smith and Murphy, 2010), both validated to reduce mitochondrial ROS in HeLaGAL cells (Fig. 4 C). As anticipated, ROS quenching abolished MDB formation (representative images of double-positive cells in Fig. 4, D and E; and quantification in Fig. 4, F i–iv). Import-competent mitochondria transferred into import-defective cells instead remained dispersed as relatively small and fragmented single mitochondrions (mean projected area of 0.528 and 0.529 µm^2^ for NACA and mitoQ, respectively) as opposed to trafficking into MDBs.

### Transferred mitochondria are ultimately degraded

**Figure 5.**
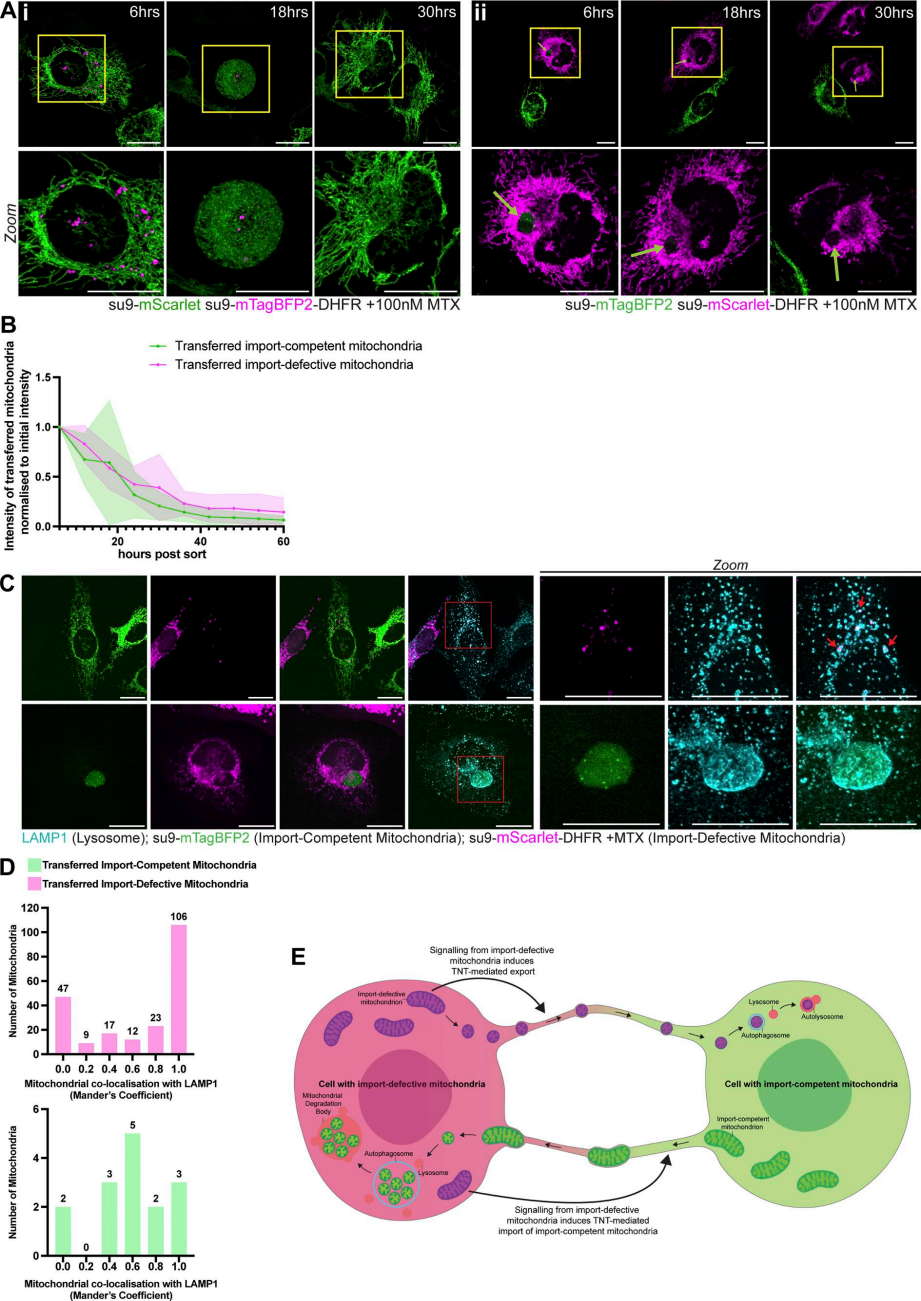
**Transferred mitochondria are ultimately degraded. (Ai)** Stills from a representative movie of transferred import-defective mitochondria (su9-mTagBFP2-DHFR +100 nM MTX) in a cell containing import-competent mitochondria (su9-mScarlet) at the indicated time points after sorting. **(Aii)** Stills from a representative movie of transferred import-competent mitochondria (su9-mTagBFP2) in a cell containing import-defective mitochondria (su9-mScarlet-DHFR +100 nM MTX) at the indicated time points after sorting. **(B)** Quantification of intensity of transferred import-defective mitochondria (su9-mTagBFP2-DHFR +100 nM MTX) normalized to intensity at 6 h after sorting (*n* = 11 cells), and quantification of intensity of transferred import-competent mitochondria (su9-mTagBFP2) normalized to intensity at 6 h after sorting (*n* = 7 cells). Lines plotted from mean normalized intensity for each time point, and area shading shows standard deviation. **(C)** Representative images of LAMP1-stained double-positive cells from a 48 h co-culture of su9-mTagBFP2 and su9-mScarlet-DHFR cell lines in the presence of MTX. Scale bars 20 μm. **(D)** Histogram of Mander’s coefficient for mitochondrial co-localization with LAMP1. *n* = 47 cells from *N* = 3 technical replicates. **(E)** Schematic of proposed model.

To understand the long-term fate of transferred mitochondria, extended live imaging was performed on double-positive cells isolated from a co-culture of import-competent and import-defective mitochondria. As noted above, both mScarlet and mTagBFP2 remain visible in the lysosome, but only the latter can be degraded (Kim and Seong, 2021; Shinoda et al., 2018; Clancy et al., 2023). Thus, when cells expressing su9-mTagBFP2-DHFR were co-cultured with those expressing su9-mScarlet, we were able to follow the fate of transferred import-defective mitochondria by virtue of their fluorescence loss (Fig. 5 A i). Conversely, a co-culture of su9-mScarlet-DHFR and su9-mTagBFP2 cell lines was used to follow the fate of transferred import-competent mitochondria within the MDBs (Fig. 5 A ii).

The fluorescence intensity of the transferred mitochondria over the 60 h imaging period was then calculated and plotted relative to the initial intensity (Fig. 5 B). Transferred import-defective mitochondria showed an intensity of 52.2% of the initial intensity at 18 h after sorting, 19.5% at 36 h, and 9.13% at 60 h. Similarly, transferred import-competent mitochondria exhibited an intensity of 41.0% of the initial intensity at 18 h after sorting, 11.6% at 36 h, and 5.38% at 60 h. These reductions in fluorescence intensity are indicative of lysosomal degradation. Co-localization of transferred import-defective and import-competent mitochondria with the lysosomal marker LAMP1 confirmed this (representative images in Fig. 5 C and quantification in D). Overall, our results show that transferred mitochondria, regardless of their fragmentation and dispersal or accumulation within MDBs, are degraded by their recipients at similar rates.

### Conclusions

We show here that bidirectional intercellular transfer of import-competent and import-defective mitochondria via TNTs ultimately leads to trans-mitophagy. We hypothesize that the different directions of traffic establish distinct, but complementary, mechanisms for the rescue of mitochondrial import failure (Fig. 5 E). On the one hand, we suppose the intercellular trafficking of import-defective mitochondria is simply for the purpose of trans-mitophagy (Davis et al., 2014). In these cells, the capacity for autophagy is likely overwhelmed, with export of compromised mitochondria serving to outsource the degradative burden to another cell, noted also elsewhere (Chakraborty et al., 2026). This process may be particularly important for cells requiring high levels of mitochondrial import (for organellar maintenance and regeneration) and thereby being prone to high levels of mitochondrial import dysfunction. Cells with low capability for mitophagy might also need to jettison their mitochondria for degradation elsewhere.

In addition to this, we suggest that transfer of import-competent mitochondria could be a response to extracellular stress signalling from cells containing import-defective mitochondria. Similar to reports elsewhere (Lin et al., 2024; Kidwell et al., 2023), the degradation of these mitochondria potentially instigates additional events in the import-defective recipients to help facilitate their recovery by an as-yet unknown mechanism. While further experiments will be required to test these possibilities, this work provides new insights into the role of canonical and noncanonical trans-mitophagy in mediating rescue of mitochondrial defects.

## Materials and methods

### Generation of constructs

Fusion proteins were assembled into a lentiviral pLVX vector at the EcoRI and XbaI sites and expressed using an EF-1α promoter. Primers used to generate constructs are listed in Table S1. The su9-mTagBFP2-DHFR gene strand was synthesized *de novo* (Eurofins), and su9-mTagBFP2 was subsequently isolated from this. su9-EGFP was isolated from su9-EGFP-DHFR, a gift from H. Needs (University of Bristol, Bristol, UK). su9-mScarlet was isolated from su9-mScarlet-DHFR, a gift from H. Needs. su9-Hyper7 was isolated from #136470; Addgene plasmid. The mCherry-Fis1 used was #182580; Addgene plasmid.

### HeLa cell culture

All cell lines were maintained in T75 ventilated flasks in humidified incubators at 37°C with 5% CO_2_. HeLa cells (Cat# CCL-2; ATCC) were “conditioned” in galactose media: DMEM [+] L-Glutamine (Gibco) supplemented with 10 mM D-Galactose (Sigma-Aldrich), 1 mM Sodium Pyruvate (Gibco), 10% FBS (Gibco) for 3 wk, washing with 1 × PBS (Gibco) every 3 days. After this point, cells were considered OXPHOS dependent and termed “HeLaGAL” cells (Aguer et al., 2011; Marroquin et al., 2007). Cells were split 1:5 every 2–3 days.

### Primary astrocyte cell culture

Primary astrocytes were isolated from embryonic day 17 Han Wistar rat pups. Pregnant Han Wistar rats (Charles River) were anaesthetized using isoflurane with pure oxygen flow and humanely killed by means of cervical dislocation, following Home Office Schedule 1 regulations. Isolated cortices were washed extensively in HBSS and dissociated by incubation with 10% (vol/vol) Trypsin-EDTA solution at 37°C for 15 min.

Cortical cells were seeded at a density of 1 × 10^7^ cells in poly-L-lysine–coated T75 flasks. Astrocytes were grown in DMEM supplemented with 10% (vol/vol) FBS, 1% P/S, and 5 mM glucose, maintained in humidified incubators at 37°C with 5% CO_2_. The media was replenished every 2–3 days, and astrocytes were passaged every 7–8 days. On day 7, microglia were removed using an orbital shaker (180 rpm for 30 min), followed by the removal of oligodendrocytes (300 rpm for 6 h). Primary astrocytes were maintained in culture for up to 4 wk.

All animal care and procedures were carried out in full compliance with University of Bristol and ARRIVE guidelines, and the UK Animals Scientific Procedures Act, 1986. In addition, all experimental protocols were approved by the University of Bristol Animal Welfare and Ethics Review Body (ethics approval number UIN: UB/23/069) panel and the Biological and Genetic Modification Safety Committee.

For mitochondrial transfer experiments, astrocytes were infected with lentivirus encoding su9-EGFP-DHFR or cox8a-DsRed. After 7–14 days of expression, co-cultures were established. Astrocytes were washed several times with PBS and dissociated with Trypsin-EDTA for 3 min at 37°C. cox8a-DsRed– and su9-EGFP-DHFR–expressing astrocytes were mixed and plated onto 1 × Geltrex-coated coverslips. Co-cultures were incubated for 24 h at 37°C with 5% CO_2_, followed by treatment with 100 nM MTX for 48 h to block mitochondrial protein import in su9-EGFP-DHFR–infected cells.

### Drug treatments

MTX CAS# 133073-73-1; 100 nM working concentration. NACA CAS# 38520-57-9; 750 μM working concentration. mitoQ CAS# 845959-50-4; 100 nM working concentration.

### Lentivirus production, transduction, and selection

Lentiviral particles were produced in HEK293T cells (Cat# CRL-3216; ATCC) using the construct of interest in the pLVX vector, alongside packaging vectors pAX2 and pMDG2, in combination with pEI transfection reagent (Cambridge Bioscience).

HEK293Ts were washed with 1 × PBS, then the PEI/DNA solution was added, and cells were incubated for 4 h at 37°C and 5% CO_2_. Following this, the PEI/DNA solution was removed and replaced with complete media.

Lentivirus was harvested after 72 h. Media was removed and spun at 4,000 rpm for 5 min to pellet dead cells, and the supernatant was then filtered through a 0.45-μm filter and added to HeLaGALs or aliquoted and stored at −80°C for future use.

Infected HeLaGALs were incubated for 72 h prior to selection. Cells were washed in 1 × PBS, and complete media containing 3 μg/ml puromycin was added. This was repeated every day until no cells were left in the uninfected control well. Remaining cells in the infected wells were grown up for experimental use.

### Transfection

HeLaGAL cells were plated and grown to 80% confluency, then transfected with 1 μg per 35-mm dish of mCherry-Fis1 plasmid (#182580; Addgene) using Lipofectamine 3000 reagent (Thermo Fisher Scientific) according to the manufacturer’s protocol. Cells were incubated for a further 24 h prior to a media change and a further 24 h incubation before experimental analysis.

### siRNA knockdown

Dharmacon ON-TARGETplus 2.0 siRNA pools used are listed in Table S2. siRNAs were resuspended in 1× siRNA Buffer (5× siRNA Buffer diluted in nuclease-free water, Dharmacon). 80% confluent HeLaGAL cells were transfected with a final concentration of 25 nM siRNA using DharmaFECT 1 Transfection Reagent, according to the manufacturer’s protocol. Transfected cells were incubated for 24 h prior to use in co-cultures.

### Western blotting

Treated cells were washed in ice-cold PBS prior to lysis with ice-cold RIPA (50 mM Tris-HCl, pH 7.4; 150 mM NaCl; 1% NP40; 0.5% Sodium deoxycholate; 0.1% SDS; Halt protease and phosphatase inhibitors 1/100 #1861280; Thermo Fisher Scientific). Lysates were clarified by pelleting at 13,200 rpm for 10 min. Supernatants were transferred to fresh tubes, and protein concentrations were determined using a DC protein assay (#500-0112; BioRAD). Samples were diluted to equal concentrations in RIPA and 2× NuPAGE sample buffer with 5% β-mercaptoethanol. Samples were then denatured at 95°C for 5 min. Protein lysates were separated on a NuPAGE 12% Bis-Tris Protein Gel and transferred onto activated PVDF membranes. Membranes were blocked for 1 h in 5% milk in PBST, then incubated with the appropriate antibody (Rabbit Anti-Human Timm23 Cat# 34822; Cell Signalling; Rabbit Anti-Human Tomm40 Cat# 55959; Cell Signalling; Mouse Anti-Human β-Actin Cat# 3700; Cell Signalling) at 4°C overnight. Membranes were washed with PBST, then probed with the appropriate secondary antibody—Alexa Fluor 680 Goat Anti-Mouse IgG (Cat# A10038; Life Technologies) or Alexa Fluor 800 Goat Anti-Rabbit IgG (Cat# W10824; Life Technologies) —in 5% milk in PBST for 1 h at room temperature. Membranes were washed with PBST, then imaged on an Odyssey Clx scanner (LICORbio).

### mitoSOX treatment

A 5 mM stock of mitoSOX Red (Invitrogen) was prepared fresh in anhydrous DMSO. Cells were incubated with 1 μM mitoSOX Red in serum-free media—DMEM [+] L-Glutamine (Gibco) supplemented with 10 mM D-Galactose (Sigma-Aldrich) and 1 mM Sodium Pyruvate (Gibco) —for 30 min at 37°C with 5% CO_2_ prior to preparation for flow cytometry.

### Flow cytometry

Cells were washed in 1 × PBS, then incubated at room temperature with Accutase (Sigma-Aldrich) for 15 min. Detached cells were pelleted by centrifugation at 300×*g* for 2 min, resuspended in ice-cold MACS buffer (1 × PBS, 5 mM EDTA, and 0.5% BSA), then incubated with DRAQ7 Dye (Invitrogen) prior to running on the BD LSRFortessa X-20 or the Agilent NovoCyte. Analysis was performed on FlowJo.

### FACS

Cells were washed in 1 × PBS, then incubated at room temperature with Accutase for 15 min. Detached cells were pelleted by centrifugation at 300×*g* for 2 min, resuspended in ice-cold MACS buffer (1 × PBS, 5 mM EDTA, and 0.5% BSA), then incubated with DRAQ7 Dye prior to cell sorting.

Cell sorting was performed on the BD FACS Aria II or the BD Influx sorters. Parental HeLaGAL cells were used to establish a gating strategy for the isolation of live singlet cells. Following this, monocultures of HeLaGAL cells expressing either su9-mScarlet-DHFR, su9-EGFP, or su9-mTagBFP2 were used to set up a four-way gating system in which the live singlet cells containing both mScarlet or EGFP could be sorted. Sorted cells were collected in 50% FBS +50% PBS with 1% Penicillin-Streptomycin (Gibco), pelleted by centrifugation at 300×*g* for 2 min, then resuspended in complete media with 1% Penicillin-Streptomycin (Gibco).

### Fluorescence microscopy

All fluorescence microscopy was performed on an Evident IX83 inverted microscope with a Yokogawa CSU-W1 spinning disk unit. Evident cellSens imaging software was used. Images were acquired using a PLAPON60XOSC2 objective (60× magnification and 1.5 numerical aperture) using either the 50-μm disk or the SoRa disk with a 3.2× magnification changer. A Hamamatsu Fusion BT sCMOS camera was used to collect images. Live-cell imaging was performed at 37°C with 5% CO_2_.

### LAMP1 staining

Cells were fixed in 4% PFA for 10 min, then washed three times in 1 × PBS prior to permeabilization with 0.1% saponin for 5 min at room temperature. Cells were blocked in 5% BSA in PBS for 1 h, then incubated with LAMP1 antibody (Mouse Anti-Human LAMP1, Cat# H4A3; DSHB ) diluted 1:1000 in 5% BSA in PBS for 1 h. Coverslips were then washed three times for 5 min in 1 × PBS, then incubated with secondary antibody (Alexa Fluor 488 Goat Anti-Mouse IgG, Cat# A21202; Life Technologies) diluted 1:400 in 5% BSA in PBS for 1 h. Coverslips were then washed a further three times for 5 min in 1 × PBS and a final wash in H_2_O prior to mounting on slides with Fluoromount-G (Invitrogen).

### DAPI staining

Cells were fixed in 4% PFA for 10 min, then washed three times in 1 × PBS and a final wash in H_2_O prior to mounting on slides with Fluoromount-G containing DAPI (Invitrogen).

### Live imaging probes

1000× stock of CellMask (Invitrogen) was diluted in complete media. Cells were incubated with this for 10 min, washed in 1 × PBS, then put into imaging media—DMEM [-] Phenol Red [+] L-Glutamine (Gibco), 10 mM D-Galactose, 1 mM Sodium Pyruvate, and 10% FBS.

### CLEM

Sorted cells were seeded onto 35-mm dishes with a no. 1.5 gridded coverslip, 14-mm glass diameter (Mattek) in complete media with 100 nM MTX, 1% P/S, and left overnight to adhere. Cells were live imaged on a Spinning disc microscope, and fluorescence images were taken along with a DIC overview of grid coordinates.

Samples were then fixed in 2.5% glutaraldehyde in 0.1 M cacodylate buffer (pH 7.3) for 30 min at room temperature. Dishes were washed in 0.1 M cacodylate buffer before post-fixing in 1% osmium and 1% potassium ferrocyanide in 0.1 M cacodylate buffer for 1 h at room temperature. Dishes were then washed in water and stained with 3% uranyl acetate for 1 h at room temperature prior to rinsing three times in water and then dehydrated through a graded ethanol series (50%, 70%, 80%, 90%, 96%, and 100%) for 5 min each. Samples were then infiltrated in Epoxy resin three times (1 h each) before polymerization at 60°C for 48 h.

Coverslips were removed by immersing dishes in liquid nitrogen, followed by boiling water. Samples were trimmed to the ROI with razor blades. 70-nm (thin) and 250-nm (thick) sections were cut on an Ultramicrotome (UC7, Leica) using a diamond knife (Diatome) and mounted onto formvar-coated slot grids.

Images were acquired at 120 kV on a Talos L120C transmission electron microscope equipped with Lab6 filament and 4K × 4K Ceta camera.

Fluorescence images were correlated with electron microscopy images using easy cell-CLEM (Paul-Gilloteaux et al., 2017) in the Icy platform (de Chaumont et al., 2012).

### Light microscopy image processing and analysis

Iterative deconvolution was performed on Cellvis software. Images were max-projected and analyzed on Fiji ImageJ (Schneider et al., 2012) using ModularImageAnalysis (Cross et al., 2024). GraphPad Prism software was used for all statistical analyses and graph design.

## Supplementary Material

Review History

Table S1 shows primer sequences.

Table S2 shows siRNA sequences.

SourceData FS3 is the source file for Fig. S3.
