# Supplementary figures and images for: Intercellular mitochondrial transfer and trans-mitophagy in response to protein import dysfunction
Source: J Cell Biol. 2026 Jul 7;225(9):e202511211. doi: 10.1083/jcb.202511211 (PMC13340441; doi:10.1083/jcb.202511211)

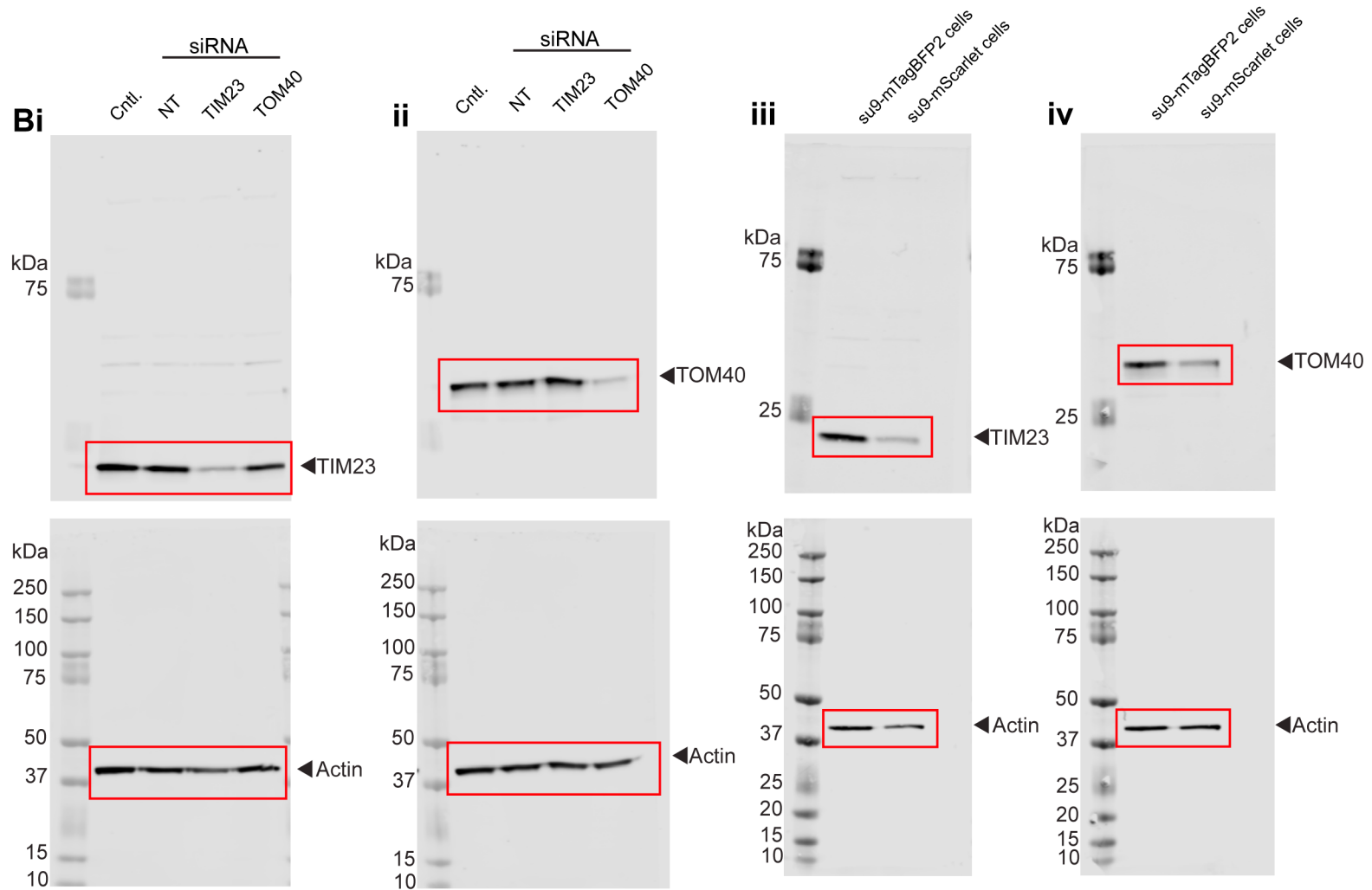

Supplement: SourceData FS3 — is the source file for Fig. S3. [file jcb_202511211_sourcedatafs3.pdf]
