# Supplementary material for: Intercellular mitochondrial transfer and trans-mitophagy in response to protein import dysfunction
Source: J Cell Biol. 2026 Jul 7;225(9):e202511211. doi: 10.1083/jcb.202511211 (PMC13340441; doi:10.1083/jcb.202511211)
Supplement: Review History [file jcb_202511211_review_history.pdf]

JCB

Intercellular mitochondrial transfer and trans-mitophagy in response to protein import dysfunction

Emily Glover, Bethan Wiseman, Celyn Dugdale, Charlotte Humphery, Lorena Sueiro Ballesteros, Lorna Hodgson, Kevin Wilkinson, and Ian Collinson

Corresponding Author(s): Ian Collinson, University of Bristol

Review Timeline:

Submission Date:	2025-11-28
Editorial Decision:	2026-01-30
Revision Received:	2026-05-01
Editorial Decision:	2026-05-28
Revision Received:	2026-06-09

Monitoring Editor: Michael Lazarou

Scientific Editor: Therese Gerbich

Transaction Report:

(Note: With the exception of the correction of typographical or spelling errors that could be a source of ambiguity, letters and reports are not edited. The original formatting of letters and referee reports may not be reflected in this compilation.)

DOI: https://doi.org/10.1083/jcb.202511211

January 30, 2026

Re: JCB manuscript #202511211

Ian Collinson
University of Bristol

Dear Prof. Collinson,

Thank you for submitting your manuscript entitled "Intercellular mitochondrial transfer and transmitophagy in response to protein import dysfunction". Your manuscript has been assessed by expert reviewers, whose comments are appended below. Although we normally employ three reviewers, in this case we have moved forward with two in the interest of not delaying your manuscript in the review process. The reviewers express potential interest in this work, however significant concerns would need to be addressed prior to publication of the manuscript in JCB.

You will see that both reviewers request validation of the results using a different method of impairing import as well as to do experiments manipulation ROS levels with antioxidants. These experiments, as well as the time course requested by reviewer two, should be included in any revision.

In order to strengthen your paper we would also highly recommend following the suggestion of reviewer #1 to quantify the ROS levels in transferred mitochondria as well as the suggestion of reviewer #2 to take a genetic approach. We recognize that these may be technically challenging, and if these experiments are not possible then please address this in your letter. We feel that the biogenesis experiments requested by reviewer #1 are beyond the scope of this work and do not need to be included.

Please let us know if you are able to address the major issues outlined above and wish to submit a revised manuscript to JCB. Note that a substantial amount of additional experimental data likely would be needed to satisfactorily address the concerns of the reviewers. The typical timeframe for revisions is three to four months. If you anticipate any difficulties in meeting this aforementioned revision time limit, please contact us and we can work with you to find an appropriate time frame for resubmission. Please note that papers are generally considered through only one revision cycle, so any revised manuscript will likely be either accepted or rejected.

If you choose to revise and resubmit your manuscript, please also attend to the following editorial points. Please direct any editorial questions to the journal office.

GENERAL GUIDELINES:
Text limits: Character count is < 40,000, not including spaces. Count includes title page, abstract, introduction, results, discussion, and acknowledgments. Count does not include materials and methods, figure legends, references, tables, or supplemental legends.

Figures: Your manuscript may have up to 10 main text figures. To avoid delays in production, figures must be prepared according to the policies outlined in our Instructions to Authors, under Data Presentation, https://jcb.rupress.org/site/misc/ifora.xhtml. All figures in accepted manuscripts will be screened prior to publication.

IMPORTANT: It is JCB policy that if requested, original data images must be made available. Failure to provide original images upon request will result in unavoidable delays in publication. Please ensure that you have access to all original microscopy and blot data images before submitting your revision.

Supplemental information: There are strict limits on the allowable amount of supplemental data. Your manuscript may have up to 5 supplemental figures. Up to 10 supplemental videos or flash animations are allowed. A summary of all supplemental material should appear at the end of the Materials and methods section.

Please note that JCB now requires authors to submit Source Data used to generate figures containing gels and Western blots with all revised manuscripts. This Source Data consists of fully uncropped and unprocessed images for each gel/blot displayed in the main and supplemental figures. For assays performed using capillary electrophoresis and/or immunoassay-based detection, authors should instead provide the electropherogram graph(s) for each experiment, plotting fluorescence/chemiluminescence intensity vs. molecular weight/size. Please be sure to provide one Source Data file for each figure gels, blots, and/or capillary electrophoresis assays along with your revised manuscript files. File names for Source Data figures should be alphanumeric without any spaces or special characters (i.e., SourceDataF#, where F# refers to the associated main figure number or SourceDataFS# for those associated with Supplementary figures). For traditional gels and blots, the lanes of the gels/blots should be labeled as they are in the associated figure, the place where cropping was applied should be marked (with a box), and molecular weight/size standards should be labeled wherever possible. For capillary electrophoresis assays, each trace in the graph should be color-coded and labeled to indicate which protein, gene, or sample is being measured

(please try to avoid red/green combinations to accommodate our color-blind readers).

Source Data files will be made available to reviewers during evaluation of revised manuscripts and, if your paper is eventually published in JCB, the files will be directly linked to specific figures in the published article.

Source Data Figures should be provided as individual PDF files (one file per figure). Authors should endeavor to retain a minimum resolution of 300 dpi or pixels per inch. Please review our instructions for export from Photoshop, Illustrator, and PowerPoint here: https://rupress.org/jcb/pages/submission-guidelines#revised

If you choose to resubmit, please include a cover letter addressing the reviewers' comments point by point. Please also highlight all changes in the text of the manuscript.

Regardless of how you choose to proceed, we hope that the comments below will prove constructive as your work progresses. We would be happy to discuss them further once you've had a chance to consider the points raised. You can contact the journal office with any questions at cellbio@rockefeller.edu.

Thank you for thinking of JCB as an appropriate place to publish your work.

Sincerely,

Michael Lazarou
Monitoring Editor
Journal of Cell Biology

Therese Gerbich
Scientific Editor
Journal of Cell Biology

Reviewer #1 (Comments to the Authors (Required)):

Reviewer Comments
The study by Glover et al. extends their previous work on intercellular mitochondrial transfer and describes distinct post-transfer fates for import-competent and import-defective mitochondria, including the identification of a previously uncharacterised compartment, termed the mitochondrial degradation body (MDB), that participates in a non-canonical form of transmitophagy of transferred import-competent mitochondria. This represents a potentially important contribution to the understanding of mitochondrial quality control and intercellular communication.

Major comments:
1. ROS levels and mitochondrial trafficking to MDBs
The authors propose that ROS levels distinguish the trafficking of transferred mitochondria toward either MDBs or canonical lysosomal pathways. The imaging is of high quality, and the model is compelling; additional experimental support would substantially strengthen the mechanistic framework:
• Direct ROS quantification in transferred mitochondria
It would be valuable to quantify ROS levels in:
(i) import competent mitochondria located in MDBs of import defective recipient cells;
(ii) mitochondria within TNTs; and
(iii) import defective mitochondria routed for transmitophagy in import competent cells.
Such data would provide direct support for ROS dependent sorting following intercellular transfer.
• Evidence for ROS alters mitochondrial trafficking fate?
Testing whether modulation of ROS (e.g., antioxidant treatment) alters mitochondrial routing or MDB formation, even in the presence of protein import defects, would help to directly test whether ROS acts as a determinant of mitochondrial trafficking fate into MDBs post-intercellular transfer.
• Downstream responses associated with mitochondrial "regeneration"
The abstract suggests that canonical and non canonical transmitophagy may cooperate to promote mitochondrial regeneration. Evaluating established downstream pathways-such as ERK activation in response to mitochondrial ROS (Kidwell et al 2023, as cited in the manuscript) or NRF2 mediated transcriptional programmes-would clarify whether recipient cells initiate compensatory biogenesis or stress adaptation responses. This point is optional since the manuscript is submitted in a Report format.
Measurements of mitochondrial biogenesis rates in cells receiving import-competent mitochondria would, however, further substantiate this model.

2. Validation across additional mitochondrial protein import-defect models
The MTX-DHFR system is appropriate and effectively induces mitochondrial protein import stress. To demonstrate that the observed phenotypes, particularly the newly reported mitochondrial trafficking to MDBs, are general consequences of impaired mitochondrial protein import, it would be important and useful to validate key findings using additional mitochondrial protein import perturbations.
• Similar analyses performed in cells treated with siRNA targeting components of the import machinery or MitoBloCK-20 (an inhibitor of the presequence pathway acting on TIM17; as in the authors' previous study) would help establish whether the observed phenotypes, especially mitochondrial trafficking to MDBs, are conserved features of protein import inhibition.
This would also strengthen the authors' conclusions - "the transfer of a large number of highly fragmented mitochondria as well as the trafficking of mitochondria into MDBs are specific features of bidirectional transfer between cells with import-competent mitochondria and cells undergoing mitochondrial import failure".

Minor Comments
• The reference cited as (10) in the text appears to be incorrect; this should refer to reference (9) in the reference list. Please also revise other references.
• The term "mitochondrial degradation bodies (MDBs)" is misspelled as "MBDs" in the abstract.
• In Figure 1Civ, it should be clearly stated what each data point represents. If these values correspond to the length of individual mitochondria within TNTs, this should be explicitly noted in the figure legend.
• Figure 3 presentation could be improved by placing the CHX and DHFR condition labels directly adjacent to the corresponding fluorescence images to facilitate linkage with the quantification shown in panel 3E.
• Page 9, line 254 contains a typographical error ("of cells 'double' was performed") that requires correction.

Reviewer #2 (Comments to the Authors (Required)):

Mitochondria import most of their proteins from the cytosol. Defects in protein import cause mitochondrial dysfunction. Cultured mammalian cells can transport mitochondria through tunneling nanotubes (TNTs). This exchange of mitochondria might play a role in signaling, in the replenishment of cells with functional mitochondria or in the removal of non-functional mitochondria, however, at present details are not clear. In this study, the authors analyzed whether mitochondrial protein import defects influence the TNT-mediated exchange of mitochondria and the fate of the transferred mitochondria in receiving cells. To this end, the authors used an established model of clogger constructs that competitively impair protein import and follow the exchange of mitochondria by fluorescence (and correlative) microscopy. They report that cells exchange import-incompetent and competent mitochondria with comparable frequencies. They identify clusters of import-competent mitochondria within import-incompetent cells which they describe as mitochondrial degradation bodies, MDBs. Moreover, they propose that the redox state of mitochondria (based on Hyper7-signals) influences transfer and mitophagy propensities. In general, this is an interesting study of high technical quality that is well-suited for the JCB, even though the setup is somewhat artificial. It is not clear whether some of the observed effects are caused by the accumulation of partially imported clogger constructs. Some aspects should still be considered.

Specific points:
1. For labeling of import-incompetent mitochondria, the authors use a su9-mScarlet-DHFR reporter. Import failure will also impair the import of this reporter protein which might therefore label non-mitochondrial structures. This is unfortunate. In order to exclude this, the authors should show co-stainings of the su9-mScarlet-DHFR reporter with outer membrane reporters that are import-independent such as GFP fusions to MIRO1 or FIS1.
2. The su9-mScarlet-DHFR reporter will accumulate as partially imported intermediate. This is presumably very different to the situation in cells with dysfunctional mitochondria where low membrane potential prevents the engagement of precursors with the import machinery. Thus, the MDBs might be artifacts from mitochondria that are decorated with partially imported precursors. This has to be excluded. The authors therefore have to show that MDBs are also found if the import failure is induced by impairment of the import machinery. MitoBlock inhibitors, TOM/TIM mutants or mtDNA depletion might be used for this purpose. Again, a MIRO1 or FIS1 reporter would be useful here.
3. The authors claim that the transfer of import-competent mitochondria into incompetent cells leads to the degradation of the received mitochondria. However, this again might be an artifact of the system as the received mitochondria will bind su9-mScarlet-DHFR reporters and therefore should rapidly be converted into import-incompetent mitochondria. A genetic system would avoid this problem as acquired mitochondria would stay functional.
4. The authors propose that cells with import-defective mitochondria send signals that might depend on the redox state of these cells. To support this hypothesis, the authors should test whether the addition of antioxidants such as NAC or glutathione influences the transfer of mitochondria.
5. The authors use a sophisticated cell sorter-based selection of cells that is nicely illustrated in Figure 2. The data would be more convincing if controls were added in which cells were analyzed after different times of co-cultivation. Initially, i.e. directly after the mixing of cells, the Q2 quadrant should be empty and get filled over time once cells have exchanged mitochondria via TNTs. This would (i) allow to judge how dynamic the exchange of mitochondria is and (ii) to estimate the background of signal that does not originate from single cells.

6. Figure 4 A shows beautiful correlation microscopy data. Unfortunately, the images are very small and blow-ups get blurry. The authors might consider showing larger sections of these images as supplemental data, also to better indicate the membrane structures in the MDBs.

Dr Michael Lazarou
Monitoring Editor *Journal of Cell Biology*

Dr Therese Gerbich
Scientific Editor *Journal of Cell Biology*

30th April, 2026

Ian Collinson

Professor of Biochemistry

School of Biochemistry
University Walk
Bristol BS8 1TD
United Kingdom

Tel.: +44 780 95-39701
E-mail: ian.collinson@bristol.ac.uk

Dear Dr Lazarou and Dr Gerbich,

We would like to thank you and the reviewers for the considerable time and effort spent evaluating our manuscript. The reviews and your guidance have been extremely valuable in helping us improve and refine the manuscript. In addition to the changes directed by the review, we have made a few other typographical corrections and improvements in style. We hope you will agree that the revised version is much better, and consider it suitable for publication in the *Journal of Cell Biology*.

As directed, we conducted additional experiments and amended the text to address the points raised. The requested experiments were successful and have indeed strengthened the paper's findings and our interpretation. This new work has been incorporated into two new sections of the results. One of them describes the knockdown of essential components of the import machinery, of the outer- (TOM40) and inner-membrane (TIM23); the results indicate that the observed non-canonical trans-mitophagy is indeed a general response to loss of import activity. The other section expands on the analysis of the causative effects of ROS, wherein we show that the exposure of import compromised cells to anti-oxidants inhibits the formation of the mitochondrial degradation bodies (MDB).

Below we have prepared a point-by-point response to your instructions and to each of the reviewers.

Sincerely

Ian Collinson, on behalf of all the authors.

Point-by-point response:

Thank you for submitting your manuscript entitled "Intercellular mitochondrial transfer and transmitophagy in response to protein import dysfunction". Your manuscript has been assessed by expert reviewers, whose comments are appended below. Although we normally employ three reviewers, in this case we have moved forward with two in the interest of not delaying your manuscript in the review process. The reviewers express potential interest in this work, however significant concerns would need to be addressed prior to publication of the manuscript in JCB.

You will see that both reviewers request validation of the results using a different method of impairing import as well as to do experiments manipulation ROS levels with antioxidants. These experiments, as well as the time course requested by reviewer two, should be included in any revision.
These were excellent suggestions, and we were very pleased with the outcome (see below for the detail).

In order to strengthen your paper we would also highly recommend following the suggestion of reviewer #1 to quantify the ROS levels in transferred mitochondria as well as the suggestion of reviewer #2 to take a genetic approach. We recognize that these may be technically challenging, and if these experiments are not possible then please address this in your letter.
We agree that these experiments would strengthen the paper however, the technical and time consuming nature of ROS quantification and taking genetic approaches to inhibit mitochondrial protein import have meant that these were unfortunately not possible in the timeframe given for review (see also below).

We feel that the biogenesis experiments requested by reviewer #1 are beyond the scope of this work and do not need to be included.
On this point we are in agreement.

Referee #1:

Reviewer Comments
The study by Glover et al. extends their previous work on intercellular mitochondrial transfer and describes distinct post-transfer fates for import-competent and import-defective mitochondria, including the identification of a previously uncharacterised compartment, termed the mitochondrial degradation body (MDB), that participates in a non-canonical form of transmitophagy of transferred import-competent mitochondria. This represents a potentially important contribution to the understanding of mitochondrial quality control and intercellular communication.

Major comments:
1. ROS levels and mitochondrial trafficking to MDBs
The authors propose that ROS levels distinguish the trafficking of transferred mitochondria toward either MDBs or canonical lysosomal pathways. The imaging is of high quality, and the model is compelling; additional experimental support would substantially strengthen the mechanistic framework:
• Direct ROS quantification in transferred mitochondria
It would be valuable to quantify ROS levels in:
(i) import competent mitochondria located in MDBs of import defective recipient cells;
(ii) mitochondria within TNTs; and
(iii) import defective mitochondria routed for transmitophagy in import competent cells.
Such data would provide direct support for ROS dependent sorting following intercellular transfer.
Excellent suggestion! We would also like to provide accurate ROS quantification to support our conjecture of ROS-dependent sorting following intercellular transfer. However, as anticipated by the editor (above), there are a number of technical challenges associated with direct ROS quantification.

We were able to deploy mitochondria-targeted Hyper7 (su9-Hyper7) to compare relative levels of the reactive oxygen species (ROS) hydrogen peroxide in the mitochondrial matrix in cells containing either import-competent or import-defective mitochondria (Figure 4A). This quantification was possible in these monocultures as the utilised fluorescent protein (FP) su9-mScarlet-DHFR has negligible impact on

Hyper7 detection. However, the cells in the co-cultures used to monitor mitochondrial transfer contained either EGFP or mTagBFP2, which both have broad emissions that bleed through into the Hyper7 detection channel. Correcting for this overlapping emission, without compromising the reliability of the data, turned out to be too technically challenging. Moreover, the time available for the revision was insufficient to identify and configure FACS and Hyper7 compatible FPs.

An alternative strategy would be to use the ROS sensitive dye mitoSOX. However, probe accumulation has been shown to be dependent on mitochondrial size, shape and mass [1]. Given the different morphologies of transferred mitochondria relative to resident networks, this would likely be unsuitable for comparing the potentially subtle differences in ROS. Additionally, as with Hyper7, careful correction of FP emission overlap would be required. Unfortunately, these challenges were also insurmountable in the time allowed for the revision.

• Evidence for ROS alters mitochondrial trafficking fate?
Testing whether modulation of ROS (e.g., antioxidant treatment) alters mitochondrial routing or MDB formation, even in the presence of protein import defects, would help to directly test whether ROS acts as a determinant of mitochondrial trafficking fate into MDBs post-intercellular transfer.
Good idea! We were able to address this experimentally in the revised manuscript (Figure 4D-F). To do so, we conducted a 48hr co-culture of su9-mTagBFP2 cells (Import-Competent) with su9-mScarlet-DHFR cells in the presence of MTX (Import-Defective) and either the general antioxidant N-Acetylcysteine amide (NACA) [2] (Figure 4D) or the mitochondria-specific antioxidant mitoQ [3] (Figure 4E). Importantly, both treatments abolish MDB formation, supporting our hypothesis that mitochondrial ROS is indeed a determinant of mitochondrial fate post-transfer.

• Downstream responses associated with mitochondrial "regeneration"
The abstract suggests that canonical and non canonical transmitophagy may cooperate to promote mitochondrial regeneration. Evaluating established downstream pathways-such as ERK activation in response to mitochondrial ROS (Kidwell et al 2023, as cited in the manuscript) or NRF2 mediated transcriptional programmes-would clarify whether recipient cells initiate compensatory biogenesis or stress adaptation responses. This point is optional since the manuscript is submitted in a Report format.
Certainly, analysis of the downstream pathways would be very informative. However, since the primary aim of our manuscript was to evaluate the fate of transferred mitochondria, we agree with the reviewer that these experiments are not essential at this stage. Given more time, we hope to address this important question in future work.

Measurements of mitochondrial biogenesis rates in cells receiving import-competent mitochondria would, however, further substantiate this model.
Again, we agree that this analysis would further support our proposed model. Unfortunately, it would not be possible to do this properly in the time permitted; particularly given the technical challenges of measuring this specifically in cells subject to mitochondrial import. Thus, we agree with the Editor that these experiments are beyond the scope of the present study.

2. Validation across additional mitochondrial protein import-defect models
The MTX-DHFR system is appropriate and effectively induces mitochondrial protein import stress. To demonstrate that the observed phenotypes, particularly the newly reported mitochondrial trafficking to MDBs, are general consequences of impaired mitochondrial protein import, it would be important and useful to validate key findings using additional mitochondrial protein import perturbations.
• Similar analyses performed in cells treated with siRNA targeting components of the import machinery or MitoBloCK-20 (an inhibitor of the presequence pathway acting on TIM17; as in the authors' previous study) would help establish whether the observed phenotypes, especially mitochondrial trafficking to MDBs, are conserved features of protein import inhibition.
This would also strengthen the authors' conclusions - "the transfer of a large number of highly fragmented mitochondria as well as the trafficking of mitochondria into MDBs are specific features of bidirectional transfer between cells with import-competent mitochondria and cells undergoing mitochondrial import failure".
This is a very good suggestion. To determine if the observed phenotypes were in response to mitochondrial import perturbation in general, we established other means to induce protein import

dysfunction. These new experiments have been included in the revision (Figure 2H and Supplementary Figure 3).

Import-Competent cells containing su9-mTagBFP2 were co-cultured with cells harbouring su9-mScarlet that had been subjected to siRNA knockdowns of TIM23 or TOM40, as an alternative way (to DHFR-MTX) to block mitochondrial import. The results show that both TIM23 and TOM40 knockdowns (compared to non-targeted siRNA) recapitulated the phenotypes observed with the DHFR-MTX system. Thus, the new data supports our conclusion that the mitochondrial degradation bodies (MDBs) are a general response to mitochondrial import dysfunction.

Minor Comments
• The reference cited as (10) in the text appears to be incorrect; this should refer to reference (9) in the reference list. Please also revise other references.
• The term "mitochondrial degradation bodies (MDBs)" is misspelled as "MBDs" in the abstract.
• In Figure 1Civ, it should be clearly stated what each data point represents. If these values correspond to the length of individual mitochondria within TNTs, this should be explicitly noted in the figure legend.
• Figure 3 presentation could be improved by placing the CHX and DHFR condition labels directly adjacent to the corresponding fluorescence images to facilitate linkage with the quantification shown in panel 3E.
• Page 9, line 254 contains a typographical error ("of cells 'double' was performed") that requires correction.
We thank the reviewer for these comments, which we have now corrected in the revised manuscript.

Reviewer #2

Mitochondria import most of their proteins from the cytosol. Defects in protein import cause mitochondrial dysfunction. Cultured mammalian cells can transport mitochondria through tunneling nanotubes (TNTs). This exchange of mitochondria might play a role in signaling, in the replenishment of cells with functional mitochondria or in the removal of non-functional mitochondria, however, at present details are not clear. In this study, the authors analyzed whether mitochondrial protein import defects influence the TNT-mediated exchange of mitochondria and the fate of the transferred mitochondria in receiving cells. To this end, the authors used an established model of clogger constructs that competitively impair protein import and follow the exchange of mitochondria by fluorescence (and correlative) microscopy. They report that cells exchange import-incompetent and competent mitochondria with comparable frequencies. They identify clusters of import-competent mitochondria within import-incompetent cells which they describe as mitochondrial degradation bodies, MDBs. Moreover, they propose that the redox state of mitochondria (based on Hyper7-signals) influences transfer and mitophagy propensities. In general, this is an interesting study of high technical quality that is well-suited for the JCB, even though the setup is somewhat artificial. It is not clear whether some of the observed effects are caused by the accumulation of partially imported clogger constructs. Some aspects should still be considered.

Specific points:
1. For labeling of import-incompetent mitochondria, the authors use a su9-mScarlet-DHFR reporter. Import failure will also impair the import of this reporter protein which might therefore label non-mitochondrial structures. This is unfortunate. In order to exclude this, the authors should show co-stainings of the su9-mScarlet-DHFR reporter with outer membrane reporters that are import-independent such as GFP fusions to MIRO1 or FIS1.
We have now analysed cells stably expressing su9-mTagBFP2-DHFR (following a 48hr incubation with MTX = trapping conditions) that were co-transfected with mCherry-Fis1. As shown in Supplementary Figure 2A of the revised manuscript, upon the import block, su9-mTagBFP2-DHFR retains almost complete co-localisation with mCherry-Fis1; confirming that it specifically labels mitochondria (and not non-mitochondrial structures).

2. The su9-mScarlet-DHFR reporter will accumulate as partially imported intermediate. This is presumably very different to the situation in cells with dysfunctional mitochondria where low membrane potential prevents the engagement of precursors with the import machinery. Thus, the MDBs might be artifacts from mitochondria that are decorated with partially imported precursors. This has to be

excluded. The authors therefore have to show that MDBs are also found if the import failure is induced by impairment of the import machinery. MitoBlock inhibitors, TOM/TIM mutants or mtDNA depletion might be used for this purpose. Again, a MIRO1 or FIS1 reporter would be useful here.
Excellent point, and very well aligned to Reviewer #1, which we have now addressed experimentally in the revised manuscript (Figure 2H and Supplementary Figure 3)—see above. The siRNA method deployed rules out that the effect is due to an accumulation of the trapped precursor su9-mScarlet-DHFR. Instead, it suggests the phenotypes we observe represent a general response to mitochondrial import inhibition.

3. The authors claim that the transfer of import-competent mitochondria into incompetent cells leads to the degradation of the received mitochondria. However, this again might be an artifact of the system as the received mitochondria will bind su9-mScarlet-DHFR reporters and therefore should rapidly be converted into import-incompetent mitochondria. A genetic system would avoid this problem as acquired mitochondria would stay functional.
Very good point. Given the time constraints for revision, we were unable to establish a genetic system to address this point. However, the experiment outlined directly above, deploying siRNA knockdown as an alternative means to compromise import, recapitulated the mitochondrial phenotypes observed using the DHFR-MTX blocking system. This rules out the potential impact of the trapping agent in the receiving cell, providing further support that the mitochondrial degradation bodies (MDBs) are a general response to mitochondrial import dysfunction.

4. The authors propose that cells with import-defective mitochondria send signals that might depend on the redox state of these cells. To support this hypothesis, the authors should test whether the addition of antioxidants such as NAC or glutathione influences the transfer of mitochondria.
Apologies for the misunderstanding, but this is not what we are proposing. We do not believe that signalling for IMT is dependent on cellular redox state. In the manuscript we have shown that IMT occurs between control co-cultures of cell lines both containing import-competent mitochondria Figure 2E-F. We are suggesting that mitochondria at the cell periphery of import-competent cells produce relatively more ROS (Figure 4A-B). Upon receiving a signal (in our case, from cells undergoing import failure), these oxidised mitochondria are therefore those that undergo IMT. We hypothesise that ROS production by import-competent mitochondria transferred into cells undergoing mitochondrial import failure cause trafficking of these mitochondria *post-transfer* into mitochondrial degradation bodies (MDBs). Indeed, we have now tested this model in our revised manuscript by demonstrating that the MDB phenotype we observe in co-cultures of import-competent and import-defective mitochondria is abolished in the presence of the antioxidants NACA or mitoQ (Figure 4D-F), suggesting a direct role for ROS in the formation of MDBs. We hope this point has now been clarified.

5. The authors use a sophisticated cell sorter-based selection of cells that is nicely illustrated in Figure 2. The data would be more convincing if controls were added in which cells were analyzed after different times of co-cultivation. Initially, i.e. directly after the mixing of cells, the Q2 quadrant should be empty and get filled over time once cells have exchanged mitochondria via TNTs. This would (i) allow to judge how dynamic the exchange of mitochondria is and (ii) to estimate the background of signal that does not originate from single cells.
We thank the reviewer for this point, which we have now addressed experimentally in our revised manuscript (Figure 2B). The co-cultivation time course confirms that the double-positive cell population increases over time. It is worth noting that GFP in the transferred mitochondria is quenched in lysosomal environment while mScarlet will be retained throughout the time course. As such, the true dynamics of mitochondrial exchange are difficult to assess using this system and this population likely represents an underestimate of the level of mitochondrial transfer.

On the point concerning the background not originating from single cells. Apologies if this was not clear in the manuscript, but the 'double-positive' population of cells are all verified single cells. This is due to the gating strategy outlined in Supplementary Figure 1A. Singlet discrimination was performed using FSC-A versus FSC-H, a standard approach in flow cytometry. Single cells show a linear relationship between FSC-A and FSC-H, while doublets or aggregates deviate from this relationship due to increased signal area. Gating on the FSC-A vs FSC-H diagonal therefore effectively excludes non-singlet events. As such, no cell doublets were considered in downstream mScarlet/GFP gating. In

addition, we have orthogonally validated this by DAPI staining of double positive cells in Supplementary Figure 2B, which shows that these cells contain a singular nucleus.

6. Figure 4 A shows beautiful correlation microscopy data. Unfortunately, the images are very small and blow-ups get blurry. The authors might consider showing larger sections of these images as supplemental data, also to better indicate the membrane structures in the MDBs.
Apologies for this. This has now been corrected in the revised manuscript.

References:

1. Murphy MP, Bayir H, Belousov V, Chang CJ, Davies KJA, Davies MJ, Dick TP, Finkel T, Forman HJ, Janssen-Heininger Y, et al.: **Guidelines for measuring reactive oxygen species and oxidative damage in cells and in vivo**. *Nature Metabolism 2022 4:6* 2022, **4**:651–662.
2. Sunitha K, Hemshekhar M, Thushara RM, Santhosh MS, Yariswamy M, Kemparaju K, Girish KS: **N-Acetylcysteine amide: a derivative to fulfill the promises of N-Acetylcysteine**. *Free Radic Res* 2013, **47**:357–367.
3. Smith RAJ, Murphy MP: **Animal and human studies with the mitochondria-targeted antioxidant MitoQ**. *Ann N Y Acad Sci* 2010, **1201**:96–103.

May 28, 2026

RE: JCB Manuscript #202511211R

Ian Collinson
University of Bristol

Dear Prof. Collinson:

Thank you for submitting your revised manuscript entitled "Intercellular mitochondrial transfer and trans-mitophagy in response to protein import dysfunction". We would be happy to publish your paper in JCB pending final revisions necessary to meet our formatting guidelines (see details below).

To avoid unnecessary delays in the acceptance and publication of your paper, please read the following information carefully.

A. MANUSCRIPT ORGANIZATION AND FORMATTING:

Full guidelines are available on our Instructions for Authors page, https://jcb.rupress.org/submission-guidelines#revised.
Submission of a paper that does not conform to JCB guidelines will delay the acceptance of your manuscript.

1) Text limits: Character count for Reports is < 20,000, not including spaces. Count includes abstract, introduction, results and discussion, and acknowledgments. Count does not include title page, figure legends, materials and methods, references, tables, or supplemental legends.

2) Figures limits: Reports may have up to 5 main text figures.

3) Figure formatting: Scale bars must be present on all microscopy images, including inset magnifications. Molecular weight or nucleic acid size markers must be included on all gel electrophoresis. Aspect ratios of images may not be altered.

4) Statistical analysis: Error bars on graphic representations of numerical data must be clearly described in the figure legend. The number of independent data points (n) represented in a graph must be indicated in the legend. Statistical methods should be explained in full in the materials and methods. For figures presenting pooled data the statistical measure should be defined in the figure legends. Please also be sure to indicate the statistical tests used in each of your experiments (either in the figure legend itself or in a separate methods section) as well as the parameters of the test (for example, if you ran a t-test, please indicate if it was one- or two-sided, etc.). Also, if you used parametric tests, please indicate if the data distribution was tested for normality (and if so, how). If not, you must state something to the effect that "Data distribution was assumed to be normal but this was not formally tested."

2B and 4B, please indicate what error bars represent in figure legend.

5) Abstract and title: The abstract should be no longer than 160 words and should communicate the significance of the paper for a general audience. The title should be less than 100 characters including spaces. Make the title concise but accessible to a general readership.

6) Materials and methods: Should be comprehensive and not simply reference a previous publication for details on how an experiment was performed. Please provide full descriptions in the text for readers who may not have access to referenced manuscripts.

7) All antibodies, cell lines, animals, and tools used in the manuscript should be described in full, including accession numbers for materials available in a public repository such as the Resource Identification Portal. Please be sure to provide the sequences for all of your primers/oligos and RNAi constructs in the materials and methods. You must also indicate in the methods the source, species, and catalog numbers (where appropriate) for all of your antibodies. Please also indicate the acquisition and quantification methods for immunoblotting/western blots.

8) Microscope image acquisition: The following information must be provided about the acquisition and processing of images:
a. Make and model of microscope
b. Type, magnification, and numerical aperture of the objective lenses
c. Temperature
d. Imaging medium
e. Fluorochromes
f. Camera make and model
g. Acquisition software

h. Any software used for image processing subsequent to data acquisition. Please include details and types of operations involved (e.g., type of deconvolution, 3D reconstitutions, surface or volume rendering, gamma adjustments, etc.).

Please provide full microscope and acquisition details for light microscopy experiments.

9) References: There is no limit to the number of references cited in a manuscript. References should be cited parenthetically in the text by author and year of publication. Abbreviate the names of journals according to PubMed.

Please adjust reference formatting and in-text citation to follow this convention.

10) Supplemental materials: There are strict limits on the allowable amount of supplemental data. Reports may have up to 3 supplemental figures. Please also note that tables, like figures, should be provided as individual, editable files. A summary of all supplemental material should appear at the end of the Materials and methods section.

11) eTOC summary: A ~40-50-word summary that describes the context and significance of the findings for a general readership should be included on the title page. The statement should be written in the present tense and refer to the work in the third person.

Please include an eTOC summary.

12) Conflict of interest statement: JCB requires inclusion of a statement in the acknowledgements regarding competing financial interests. If no competing financial interests exist, please include the following statement: "The authors declare no competing financial interests." If competing interests are declared, please follow your statement of these competing interests with the following statement: "The authors declare no further competing financial interests."

13) ORCID IDs: ORCID IDs are unique identifiers allowing researchers to create a record of their various scholarly contributions in a single place. Please note that ORCID IDs are now *required* for all authors. At resubmission of your final files, please be sure to provide your ORCID ID and those of all co-authors.

14) A separate author contribution section following the Acknowledgments. All authors should be mentioned and designated by their full names. We encourage use of the CRediT nomenclature.

Please use full author names in this section.

Please note that JCB now requires authors to submit Source Data used to generate figures containing gels and Western blots with all revised manuscripts. This Source Data consists of fully uncropped and unprocessed images for each gel/blot displayed in the main and supplemental figures. For assays performed using capillary electrophoresis and/or immunoassay-based detection, authors should instead provide the electropherogram graph(s) for each experiment, plotting fluorescence/chemiluminescence intensity vs. molecular weight/size. Please be sure to provide one Source Data file for each figure gels, blots, and/or capillary electrophoresis assays along with your revised manuscript files. File names for Source Data figures should be alphanumeric without any spaces or special characters (i.e., SourceDataF#, where F# refers to the associated main figure number or SourceDataFS# for those associated with Supplementary figures). For traditional gels and blots, the lanes of the gels/blots should be labeled as they are in the associated figure, the place where cropping was applied should be marked (with a box), and molecular weight/size standards should be labeled wherever possible. For capillary electrophoresis assays, each trace in the graph should be color-coded and labeled to indicate which protein, gene, or sample is being measured (please try to avoid red/green combinations to accommodate our color-blind readers).

Source Data files will be made available to reviewers during evaluation of revised manuscripts and, if your paper is eventually published in JCB, the files will be directly linked to specific figures in the published article.

Source Data Figures should be provided as individual PDF files (one file per figure). Authors should endeavor to retain a minimum resolution of 300 dpi or pixels per inch. Please review our instructions for export from Photoshop, Illustrator, and PowerPoint here: https://rupress.org/jcb/pages/submission-guidelines#revised

Journal of Cell Biology now requires a data availability statement for all research article submissions. These statements will be published in the article directly above the Acknowledgments. The statement should address all data underlying the research presented in the manuscript. Please visit the JCB instructions for authors for guidelines and examples of statements at (https://rupress.org/jcb/pages/editorial-policies#data-availability-statement).

B. FINAL FILES:

Please upload the following materials to our online submission system. These items are required prior to acceptance. If you have any questions, contact JCB's Managing Editor, Lindsey Hollander (lhollander@rockefeller.edu).

-- An editable version of the final text (.DOC or .DOCX) is needed for copyediting (no PDFs).

-- High-resolution figure and MP4 video files: See our detailed guidelines for preparing your production-ready images, https://jcb.rupress.org/fig-vid-guidelines.

-- Cover images: If you have any striking images related to this story, we would be happy to consider them for inclusion on the journal cover. Submitted images may also be chosen for highlighting on the journal table of contents or JCB homepage carousel. Images should be uploaded as TIFF or EPS files and must be at least 300 dpi resolution.

It is JCB policy that if requested, original data images must be made available to the editors. Failure to provide original images upon request will result in unavoidable delays in publication. Please ensure that you have access to all original data images prior to final submission.

The license to publish form must be signed before your manuscript can be sent to production. A link to the license to publish form will be sent to the corresponding author only. Please take a moment to check your funder requirements before choosing the appropriate license.

Additionally, JCB encourages authors to submit a short video summary of their work. These videos are intended to convey the main messages of the study to a non-specialist, scientific audience. Think of them as an extended version of your abstract, or a short poster presentation. We encourage first authors to present the results to increase their visibility. The videos will be shared on social media to promote your work. For more detailed guidelines and tips on preparing your video, please visit https://rupress.org/jcb/pages/submission-guidelines#videoSummaries.

Thank you for your attention to these final processing requirements. Please revise and format the manuscript and upload materials within 14 days. If you need an extension for whatever reason, please let us know and we can work with you to determine a suitable revision period.

Please contact the journal office with any questions at cellbio@rockefeller.edu.

Thank you for this interesting contribution, we look forward to publishing your paper in Journal of Cell Biology.

Sincerely,

Michael Lazarou
Monitoring Editor
Journal of Cell Biology

Therese Gerbich
Scientific Editor
Journal of Cell Biology

Reviewer #1 (Comments to the Authors (Required)):

The authors have satisfactorily addressed the two key points raised in the initial review by providing additional evidence supporting ROS as a determinant of mitochondrial fate following intercellular transfer and by implementing an independent approach to perturb mitochondrial protein import via siRNA-mediated knockdown of TIM23 and TOM40 which further supports mitochondrial trafficking to MDBs as a general response to defects in mitochondrial protein import. I support the publication of this study.

Reviewer #2 (Comments to the Authors (Required)):

The authors adequately addressed the points raised on the previous submission. I fully support the publication of this interesting study in its present form.
